# Associations between emotional reactivity to stress and adolescent substance use: Differences by sex and valence

**DOI:** 10.1002/smi.3420

**Published:** 2024-05-23

**Authors:** Danny Rahal, Julienne E. Bower, Michael R. Irwin, Andrew J. Fuligni

**Affiliations:** 1 Department of Psychology, University of California, Santa Cruz, California, USA; 2 Department of Psychology, University of California, Los Angeles, California, USA; 3 Department of Psychiatry and Biobehavioral Sciences, University of California, Los Angeles, California, USA; 4 University of California, Norman Cousins Center for Psychoneuroimmunology, Los Angeles, California, USA

**Keywords:** adolescence, daily diary, drug use, emotion response, interpersonal stress

## Abstract

Although stress is often related to substance use, it remains unclear whether substance use is related to individual differences in how adolescents respond to stress. Therefore the present study examined associations between substance use and daily emotional reactivity to stress within a year across adolescence. Adolescents (*N* = 330; *Mage* = 16.40, *SD* = 0.74 at study entry; *n* = 186 female; *n* = 138 Latine; *n* = 101 European American; *n* = 72 Asian American; *n* = 19 identifying as another ethnicity including African American and Middle Eastern) completed a longitudinal study, including three assessments between the 10th grade and 3-years post-high school. At each assessment, participants reported frequency of alcohol and cannabis use and the number of substances they had ever used. They also completed 15 daily checklists, in which they reported the number of daily arguments and their daily emotion. Multilevel models suggested that more frequent alcohol and cannabis use were related to attenuated positive emotional reactivity to daily stress (i.e., smaller declines in positive emotion on days when they experienced more arguments) for both male and female adolescents. Associations for negative emotional reactivity to stress varied by sex; more frequent alcohol use and use of more substances in one’s lifetime were related to greater anxious emotional reactivity to stress among female adolescents, whereas more frequent alcohol and cannabis use and higher lifetime substance use were related to attenuated depressive emotional reactivity to stress among male adolescents. Taken together, substance use was related to emotional reactivity to daily stress within the same year during adolescence, although associations differed by valence and adolescent sex.

## 1 | INTRODUCTION

Substance use tends to increase during middle to late adolescence, or the period spanning from puberty onset to age 24 (Sawyer et al., 2018), potentially due to heightened exposure and social pressure to use (Gallegos et al., 2021; Miech et al., 2020). Late adolescence includes the transition from high school, during which youth continue to show heightened substance use that is related to stress and emotional distress (Danzo et al., 2021; Hoffmann, 2016; Neppl et al., 2023; Stone et al., 2012). Frequent substance use during adolescence is related to greater risk for problematic substance use and poorer mental health later in adulthood (e.g., Magee & Connell, 2021; McCambridge et al., 2011; Merrin & Leadbeater, 2018; Miettunen et al., 2014).

The developmental psychopathology framework highlights characterizing multiple levels of analysis, including individuals’ daily experiences, and to address the pathways by which certain youth are positioned for heightened psychopathology risk (Cicchetti & Cohen, 1995; Cichetti & Rogosch, 2002). Youth are often motivated to use due to social stress and beliefs that substances can improve emotion (Kuntsche et al., 2015), and polysubstance use is related to greater distress concurrently across adolescence (Felton et al., 2015). In line with experimental evidence relating substance use to stress and biological responses to stress (Chaplin et al., 2018; Rahal et al., 2023), frequent use may confer risk for later use by impacting how youth respond to stress in daily life, termed their emotional reactivity to stress. Greater reactivity to stress has been related to psychopathology including depressive symptoms (Bai et al., 2020), with emerging evidence that associations may differ by sex (Chaplin et al., 2019). However, few studies have assessed whether substance use can modulate how youth respond to subsequent day-to-day stressors. Therefore the present study examined how use of substances is related to differences in daily emotional responses to interpersonal stress, as both exaggerated and attenuated emotional responses to stress are related to psychopathology including depressive symptoms, and whether associations differed by sex.

### 1.1 | Emotional reactivity in adolescence

Adolescents show heightened emotional intensity and fluctuations in emotion compared to adults (Larson et al., 2002; Maciejewski et al., 2015). Neurobiological development following puberty onset can promote sensitivity to both rewarding and threatening stimuli, and adolescents’ emotional well-being tends to improve after positive peer and family interactions and worsen following negative interactions (Steinberg et al., 2018). Adolescents develop strategies for regulating emotion following stress and consequently show attenuated fluctuations in emotion as they age (Maciejewski et al., 2015; Steinberg et al., 2018). Youth can show heightened emotional fluctuations due to heightened reactivity or difficulties regulating a response (Bradley, 2003). Emotional reactivity refers to an individual’s predisposition for mounting an emotional response to a stimulus and is fundamentally distinct from emotion regulation, which refers to the process by which individuals attempt to alter the intensity and duration of their emotion (Gross, 1998; Reeck et al., 2016), although individuals might be able to adjust their responses to a stimulus when using regulatory strategies and therefore might show lower emotional reactivity.

An individuals’ capacity for responding to stressors (i.e., emotional reactivity to stress) may be related to health in ways that are distinct from their responses to other social or rewarding daily experiences. Stressors such as conflict tend to elicit intense increases in negative emotion and decreases in positive emotion, such that larger responses to daily stressors can gradually degrade health (Almeida, 2005). Emerging evidence suggests that greater emotional reactivity to negative stimuli is associated with greater depressive symptoms in children and adults (e.g., Bai et al., 2020; Herres et al., 2016), suggesting that fluctuations in emotion following daily stressful experiences may be pivotal for health. Therefore, emotional reactivity to stress may be an important but understudied aspect of daily emotion processes that is related to substance use.

### 1.2 | Substance use and emotional reactivity to stress

Substance use and emotional reactivity to stress could be related across adolescence due to bidirectional associations (Weiss et al., 2017). Both acute and regular, intense use of substances can alter psychobiological stress responses (Wemm & Sinha, 2019). Acute use of substances, most notably alcohol, in social situations can promote positive emotion and emotional stability on a given day for young adults (De Leon et al., 2020; Sayette et al., 2012), as well as dampen stress responses by disrupting negative cognitive appraisals (Levenson et al., 1980; Sayette, 1993). However, frequent use can also contribute to less stable emotions and more stressful interpersonal and physical consequences that elicit negative emotion (e.g., De Leon et al., 2020; Jones et al., 2020). For instance, disagreements regarding substance use elicit distress in adult couples (Rodriguez et al., 2014), and disagreements related to discordant use and repeated consequences of substance use may similarly elicit daily stressors by straining adolescents’ relationships with friends and family (Boman et al., 2013; Jones et al., 2020). Frequent substance use and experimentation in adolescence may carry over to alter how youth emotionally react to daily stressors, irrespective of their use on a given day, although research has not empirically tested this.

Given sex differences in substance use aetiology and motives (Danzo et al., 2021; Kuntsche et al., 2015), as well as socialization of responses to stress, it has been posited that pathways relating emotional reactivity to stress and substance use differ by sex (Chaplin et al., 2018). Male youth with attenuated emotional responses to stress may not be stimulated by daily experiences and therefore pursue risky behaviours including substance use, in line with reinforcement sensitivity theory and sensation seeking models of substance use (Zuckerman, 2007). In turn, exaggerated responses to stress may suggest difficulties coping with stress for female youth, which could thereby relate to one’s propensity for psychopathology and substance use. It is possible that substance use may similarly contribute to difficulties with stress sensitivity, which may manifest as attenuated emotional responses to stress in male adolescents and exaggerated responses to stress in female adolescents.

Although few studies have tested associations with emotional reactivity to stress, studies have related substance use to other aspects of the stress response. Both exaggerated and attenuated psychobiological responses (i.e., cortisol, autonomic nervous system responses) to laboratory-based social stress are associated with more frequent and polysubstance use later in adolescence (e.g., Evans et al., 2016; Rahal et al., 2023). One study found that harsher parenting was related to greater neural responses to negative stimuli in female adolescents, which were related to substance use, versus blunted neural responses in male adolescents (Chaplin et al., 2019). These studies have been limited to laboratory-based stressors, and similar associations may emerge between substance use and emotional reactivity to stress in daily life, which may be easier for clinicians to evaluate than biological or neural responses and deepen our understanding of how these processes carryout in daily life in line with a developmental psychopathology perspective. One survey-based study found that college students who reported generally experiencing greater immediate and sustained emotional reactivity to stress also reported greater tobacco use (Dvorak & Simons, 2008). Use of daily experience sampling, which is less susceptible to self-report bias than surveys, is needed to clarify associations between substance use and emotional reactivity to daily stress.

### 1.3 | Present study

Motivated by a developmental psychopathology framework (Cicchetti & Cohen, 1995; Cichetti & Rogosch, 2002), the present study investigated whether substance use was related to emotional reactivity to daily stress among male and female adolescents. We specifically assessed whether individuals who use more frequently over the past year or use more substances show differences in their daily emotional reactivity to stress, as opposed to testing daily associations between emotional processes and substance use, due to aspects of the study design. Adolescents from the greater Los Angeles area completed up to three assessments over 6 years, for which they reported whether they had ever used varied substances and how frequently they had used alcohol and cannabis, licit substances in California at the time of study that are also the most commonly used substances nationally and in California during adolescence (Austin et al., 2016; Miech et al., 2020), over the past year. Participants also reported daily arguments as one common interpersonal stressor and their positive, anxious, and depressive emotion each night for 15 days. Whereas prior studies have examined self-reported stress responses and psychobiological reactivity to acute stress (Dvorak & Simons, 2008; Rahal et al., 2023), we examined emotional responses to daily stressors as an ecologically valid indicator of daily stress processes that is less prone to self-report bias than metacognitive surveys regarding one’s general predisposition for emotional reactivity. Emotional reactivity to stress in daily approaches refers to the daily linkage between experiencing a daily stressor such as an argument and daily emotion (Bolger & Zuckerman, 1995; Cohen et al., 2005). Analytically, this approach contextualizes whether daily emotion differs between stressful days (i.e., days with more stressors) versus stress-free days (days with fewer or no stressors). Associations were tested within a year because research has not characterized associations between substance use daily emotional reactivity to arguments. Evidence for within-year associations across adolescence can inform the development of prospective studies testing mechanisms over time.

To provide a robust test of how substance use relates to emotional reactivity to daily stress, we examined different aspects of daily emotion and substance use. We examined positive emotion because it is fundamentally distinct from negative emotion and independently related to health behaviours including substance use (Dora et al., 2023; Kuntsche et al., 2015; Pressman et al., 2019; Russell, 1980) and examined anxiety and depressive emotion as distinct aspects of developmental psychopathology. We hypothesized that adolescents would report lower positive emotion and higher anxious and depressive emotion on days when they have more arguments, in line with the extant literature (Almeida, 2005), and that these daily associations would differ by substance use.

We also applied a developmental lens to examine risky substance use in adolescence. Frequency of alcohol use was the primary outcome because alcohol use is common and related to differences in stress responses (De Leon et al., 2020; Kuntsche et al., 2015; Sayette, 1993; Sayette et al., 2012). Use was also operationalized with respect to frequency of cannabis use as well as lifetime number of substances used, a common metric for accounting for illicit substance use. Greater experimentation with substances suggests that youth are exposed to various substances in potentially risky environments, in addition to alcohol in the home (e.g., Goldenson et al., 2017; Gustavson et al., 2017; McLarnon et al., 2014), and is a risk factor for greater use in adulthood (e.g., Merrin et al., 2018; Merrin & Leadbeater, 2018). Just as youth can discontinue alcohol use from adolescence to adulthood, use of multiple substances is not deterministic but is consistently related to poorer outcomes on average including lower rates of high school completion and more frequent substance use in adulthood (Kelly et al., 2015; Merrin & Leadbeater, 2018). Taken together, these models provide a nuanced test of how aspects of substance use relate to emotional reactivity to daily stress.

Greater substance use (i.e., more frequent use of alcohol and cannabis over the past year, use of more substances in one’s lifetime) was hypothesized to be associated with attenuated emotional reactivity to stress with respect to positive, anxious, and depressive emotion in male adolescents, and greater emotional reactivity to stress among female adolescents in line with posited mechanisms (Chaplin et al., 2018). We assessed associations at each year of assessment, with participant’s reports of substance use for that year statistically modelled as modulating the daily association between arguments and emotion (see Figures S1-S2 for a visual depiction of the study design and analytic modelling).

## 2 | METHOD

### 2.1 | Participants

The larger study included 350 total participants from the greater Los Angeles area (55.8% female; *n* = 147 Latine, 42.0%; *n* = 106 European American, 30.3%; *n* = 75 Asian American, 21.4%; *n* = 22 identifying as another ethnicity including African American and Middle Eastern, 6.3%; median annual income of $65,000). Primary caregivers reported the highest level of education achieved by each parent (1 = *some elementary school;* 2 = *completed elementary school;* 3 = *some junior high school;* 4 = *completed junior high school;* 5 = *some high school;* 6 = *graduated from high school;* 7 = *trade or vocational school;* 8 = *some college;* 9 = *graduated from college;* 10 = *some medical, law, or graduate school;* 11 = *graduated from medical, law, or graduate school*). For youth in two-parent households, parental education was averaged across both parents. Across the sample, 18.5% of participants had parents who did not graduate from high school, 16.4% had parents who graduated high school, 23.8% had parents who graduated from a trade or vocational school, 20.5% had parents who completed some other form of college, and 20.8% had parents who graduated from college or higher education as their highest form of education.

This study used an accelerated longitudinal design, including two cohorts staggered 1 year apart and followed across three waves, each 2 years apart (Figure S1). During the first wave of the study, 316 participants were recruited from 10th and 11th grade classrooms at four public high schools in the greater Los Angeles area through in-class presentations, mailings, and flyers from October 2011 to June 2012. Participants then had the option to complete additional waves of data collection two (73.1%) and four years later (64.3%). Because of attrition between the first and second waves, an additional 34 participants (26 12th graders and eight students who were 1-year post-high school) were recruited at the second wave. These participants did not differ from adolescents recruited at the first wave with respect to ethnicity, college attendance, or income, all *p*s > 0.20. By incorporating two cohorts 1 year apart, with waves staggered 2 years apart, the study included data from all years from 10th grade to 3 years post-high school. This study design enabled us to examine associations between substance use and emotional reactivity to stress in a given year throughout adolescence.

To be included in the present study, participants needed to complete reports of substance use in the survey and to complete the daily checklist protocol in at least one wave. They also needed to have provided data regarding their age, sex, and parents’ education. This resulted in 330 of the 350 participants being retained in analyses (56.4% female; *n* = 138 Latine, 41.8%; *n* = 101 European American, 30.6%; *n* = 72 Asian American, 21.8%; *n* = 19 identifying as another ethnicity including African American and Middle Eastern, 5.8%; median annual income of $65,000). These participants did not differ from the full sample with respect to sex, ethnicity, parents’ education, daily emotion, daily stress, or substance use, all *p*s > 0.30.

#### 2.1.1 | Missing data analyses

Participants completed 658 total waves (*M* = 1.99, *SD* = 0.85) and 8773 daily observations (*M* = 14.33, *SD* = 4.71 out of 15 possible per wave; *M* = 29.58, *SD* = 11.85 per participant). For each participant, we calculated the percentage of waves completed out of three possible waves if they initially entered in the first wave of the study or out of two possible waves if they initially entered in the second wave of the study. We tested whether participation in the study differed by sex using a *t*-test; ethnicity using ANOVA; parents’ education using correlation; substance use measures, daily emotion, and daily stressors using multilevel models with percentage of possible waves as the predictor, controlling for age. Participation did not differ by sex, substance use, daily emotion, or daily stressors, all *p*s > 0.05. ANOVA indicated ethnic differences in participation, *F* (326,3) = 4.17, *p* = 0.0064, and Tukey’s post-hoc tests indicated that Asian Americans participated in a lower percentage of possible waves than all other ethnic groups; *M* = 0.58, *SD* = 0.28 for Asian Americans; *M*s = 0.71–0.73, *SD*s = 0.27–0.28 for other ethnic groups. Lastly, higher parental education was weakly correlated with higher participation, *r*(328) = 0.12, *p* = 0.03.

#### 2.1.2 | Cohort differences

We tested for differences in participation and demographic information by cohort (10th vs. 11th grade at study entry). There were no differences in participation, ethnicity, sex, family income, parents’ education, by whether their mother versus another family member was the caregiver, the schools that students attended, family size, frequency of alcohol or cannabis use, lifetime use, or average levels of emotion at study entry by cohort, all *p*s > 0.05. The only tested variable that significantly differed by cohort was age, as expected, *p* < 0.001.

#### 2.2 | Procedures

At each assessment, research staff visited participants’ homes. Both adolescents and a primary caregiver completed online psychosocial questionnaires using an iPad or laptop, and each earned $50, $75, and $120 at each of the three assessments. Participants reported their sex and ethnicity at study entry, and all participants identified as either male or female. Caregivers also reported family income and parents’ education as part of this survey. All procedures were approved by the University of California, Los Angeles Institutional Review Board. Participants had the option to complete a 2-week daily protocol in which they reported whether they experienced various daily events and the extent to which they had felt positive and negative emotion at bedtime each night for 15 nights. They received an electronic stamper to mark the time at which they completed each checklist. The vast majority of checklists were completed in a timely manner (98%). Participants were given paper checklists during the in-person visit and instructed to begin the daily protocol the following day in order to minimize time between the survey and checklists. Despite the slight lag, daily checklists are ecologically valid and intended to capture experiences that are representative of that individual’s daily life beyond the designated period (Shiffman, 2009).

### 2.3 | Substance use measures

#### 2.3.1 | Frequency of alcohol and cannabis use

As part of the psychosocial survey, participants reported whether they had ever used any of the following seven substances: cigarettes, alcohol, cannabis, cocaine, crystal meth, illegal drugs, or any prescription drugs without a valid prescription. If participants had ever drunk alcohol, they reported how often they had at least one drink of alcohol over the past year using a 10-point scale (1 = *Never in the past year,* 2 = *1 or 2 days in the past year,* 3 = *3 to* 11 *days in the past year,* 4 = 1 *day a month,* 5 = 2 *to* 3 *days a month,* 6 = 1 *day a week,* 7 = 2 *days a week,* 8 = 3 *to* 4 *days a week,* 9 = 5 *to* 6 *days a week,* 10 = *Every day*). Participants who had never drank alcohol were coded as never using it in the past year (54.7% at first wave, 38.4% at second wave, 21.5% at third wave). If participants ever had used cannabis, they reported how many days they used cannabis over the past year on the same scale. Participants who had never used were coded as never using in the past year (75.6% at first wave, 59.8% at second wave, 46.8% at third wave). Both items were taken from the Youth Risk Behaviour Surveillance System, a large-scale national dataset. Substance use rates are comparable to prior studies of youth (e.g., Miech et al., 2020).

#### 2.3.2 | Lifetime substance use

Because rates of illicit substances were low within the sample, precluding assessment of frequency of illicit use, we calculated lifetime use as the number of substances a participant had ever used (0 = never used any substances, 7 = used all seven substances in their lifetime). Studies with similar measures have found that higher lifetime substance use in adolescence predicts more frequent use in adulthood (Merrin et al., 2018; Merrin & Leadbeater, 2018).

### 2.4 | Daily checklist measures

#### 2.4.1 | Daily stress

Each day, adolescents reported whether they: argued with their mother or father about something, argued with another family member about something, had an argument with a close friend or partner, or had an argument or were punished by an adult at school (0 = no, 1 = yes). Drawing from the daily stress process model (Almeida, 2005), we selected these items because interpersonal arguments and tensions constitute the domain of stressors in the Daily Inventory of Stressful Experiences that people experience most frequently in daily life (Almeida et al., 2002). Prior studies of adults have used similar items to index emotional reactivity to interpersonal arguments (e.g., Birditt et al., 2011). A sum was calculated for each day for each participant (0 = no stressors, 4 = all four stressors), with higher values indicating more daily stressors. We assessed arguments as a common, intense daily stressor using checklists, in line with daily approaches to measuring emotional reactivity to stress (e.g., Almeida, 2005; Bai et al., 2020; Bolger & Zuckerman, 1995). Greater emotional reactivity to arguments is thought to indicate an individual’s predisposition for emotionally responding to common stressors throughout the day.

#### 2.4.2 | Daily emotion

Participants reported their daily emotion using items from the Profile of Mood States (POMS; McNair et al., 1989) and Positive and Negative Affect Schedule (PANAS; Watson et al., 1988). Using a scale from 1 (*not at all*) to 5 (*extremely*), adolescents reported how much they experienced positive emotion (*interested, excited, cheerful, enthusiastic, attentive*), anxious emotion (*worried, on edge, uneasy, nervous*), and depressive emotion (*discouraged, hopeless, sad*) throughout the day. These scales have been used in previous studies of adolescents (e.g., Yip & Fuligni, 2002). Subscales showed moderate reliability across items each day (αs=0.65–0.81, Table S1). Participants reported moderate positive emotion and low negative emotion in line with prior samples of adolescents (Allan et al., 2015).

### 2.5 | Analytic plan

All models were tested in Stata 16.1 in a multilevel framework. See Figure S2 for a visual depiction of analytic models. We used multilevel models to examine demographic differences in primary study variables: positive emotion, anxious emotion, depressive emotion, daily stress, frequency of alcohol use, frequency of cannabis use, and lifetime substance use. Multilevel models assume normality of residuals at each level of analysis, do not impose distributional assumptions on predictors, and are generally robust to slight deviations from assumptions (Schielzeth et al., 2020). Models used maximum likelihood to account for missing data and allowed for all participants who completed at least one wave of data to be incorporated in the analysis. Re-testing models with restricted maximum likelihood and Bayesian estimates did not change the pattern of results. Three-level models with days (Level 1) within waves (Level 2) within adolescents (Level 3) were used for daily emotion, and two-level models with waves (Level 1) nested within adolescents (Level 2) were used for substance use measures.

Next, we tested whether individuals who used more frequently and used more substances showed differences in their emotional reactivity to stress across days. We could not assess how substance use related to emotional reactivity on a given day because we measured substance use each wave rather than each day, but we leveraged estimates of emotional reactivity across days to test associations between substance use and emotional reactivity to stress within a given year. Three-level models with days (Level 1) nested within years (Level 2) within adolescents (Level 3) were used to model emotional reactivity, with separate models for positive, anxious, and depressive emotion. Number of stressors was reported daily (Level 1) and centered at the participant’s mean at that age, and this coefficient represented the predicted difference in adolescents’ emotion on days when they experienced more stressors relative to their mean level at that age. Daily stressors were included as a random slope in all models, and the random slope of the association between daily arguments and end-of-day emotion measures individual differences in emotional reactivity. This technique for modelling individual differences in emotional reactivity, with respect to daily linkages between daily stressors and emotion reported at the end of the day, has been extensively used (e.g., Almeida, 2005; Herres et al., 2016). Although stressors could theoretically occur at any point in the day and the end-of-day emotion is likely affected by varied daily experiences, this technique leverages the multiple daily reports to reliably measure individual differences in the magnitude of this linkage.

To determine whether substance use was related to emotional reactivity to stress, models tested the cross-level two-way Substance Use (Level 2) × Daily Stress (Level 1) interaction as a predictor of emotion (Equation 1). Substance use measures were reported at each study assessment, and values were grand mean-centered. Separate models were tested for frequency of alcohol use over the past year, frequency of cannabis use over the past year, and lifetime substance use. Significant interactions would indicate that the degree to which daily stress related to emotion (i.e., the magnitude of the association between daily stress and emotion) varied by substance use. Interactions were probed at levels of substance use (never used, used 3–11 days, used 2–3 days/month in the past year for frequency measures; 0, 2, and 4 substances for lifetime substance use). The potentially arbitrary values of the mean and the mean ± one standard deviation were rounded to concrete scale values to facilitate interpretation, clinical relevance, and replicability of findings (Finsaas & Goldstein, 2021), and result in a nearly identical pattern of associations. Regions of significance were also identified using the Johnson–Neyman technique (Johnson & Fay, 1950). Models were repeated including the Age × Daily Stress interaction to avoid biasing the Substance Use × Daily Stress interaction, which resulted in a nearly identical pattern of results. The Age × Daily Stress interaction was consistently nonsignificant, suggesting that daily emotional reactivity to stress did not differ by participants’ age, and therefore omitted from presented results.

Because this is among the first studies of emotional reactivity to stress and substance use, we were primarily interested in how these variables relate to each other at a given year. Models tested associations at a given time point and utilized the multiple assessments to test associations within the same year across adolescence. Although associations may be bidirectional, substance use was the statistical predictor in models because there were several observations of emotion across days per wave. We therefore statistically modelled substance use (Level 2) as the predictor and emotion (Level 1) as the outcome, in line with past studies (e.g., Bai et al., 2020; Rahal et al., 2022, 2023; Shirtcliff & Essex, 2008). This approach includes all observations within the analysis by testing daily emotion as the outcome, accurately estimates residuals, and does not assume normality of predictor variables (i.e., substance use).

Equation 1 (i = a given day, j = a given wave, k = a given individual, u = differences by a given wave for a given individual, r = differences for a given individual):


$$L1:{\hat{\text{Emotion}}}_{ijk}=\beta_{0jk}+\beta_{1jk}\left({\text{Argument}}_{ijk}\right)\;\;+\beta_{2jk}\left({\text{Previous}\;{\text{Day}}^{\prime }\text{s}\;\text{Emotion}}_{ijk}\right)\;L2:\beta_{0jk}=\gamma_{00k}+\gamma_{01k}\left({\text{Substance}\;\text{Use}}_{jk}\right)+\gamma_{02k}\left({\text{Age}}_{jk}\right)+u_{0jk}\;\;\beta_{1jk}=\gamma_{10k}+\gamma_{11k}\left({\text{Substance}\;\text{Use}}_{jk}\right)+u_{1jk}\;\;\beta_{2jk}=\gamma_{20k}+u_{2jk}\;\text{L}3:\gamma_{00k}=\pi_{000}+\pi_{001}\left({\text{Substance}\;\text{Use}}_{k}\right)+\pi_{002}\left({\text{Age}}_{k}\right)\;\;+\;\pi_{003}(\text{Female})+\pi_{004}(\text{Asian}\;\text{American})\;\;+\;\pi_{005}(\text{European}\;\text{American})\;\;+\;\pi_{006}(\text{Different}\;\text{Ethnic}\;\text{Backgrounds})\;\;+\;\pi_{007}(\text{Parental}\;\text{Education})+r_{00k}\;\;\gamma_{01k}=\pi_{010}+r_{01k}\;\gamma_{10k}=\pi_{100}+\pi_{101}\left({\text{Substance}\;\text{Use}}_{k}\right)+r_{10k}\;\;\gamma_{11k}=\pi_{110}+r_{11k}$$

Models then tested whether associations between emotional reactivity to stress and substance use differed by sex. Models were repeated including a three-way Sex (Level 3) × Substance Use (Level 2) × Daily Stress interaction (Level 1; Equation 2). Significant three-way interactions would suggest that the Substance Use × Daily Stress interaction (i.e., the index of the association between emotional reactivity to stress and substance use) differed between male and female adolescents. In line with existing guidelines (Curran et al., 2004), we decomposed a three-way interaction by estimating the two-way interaction at each level of the third variable (sex; dummy-coded 0 = male, 1 = female). This approach is needed because a significant three-way interaction could emerge despite neither two-way interaction being significant. This approach revealed whether the two-way interaction was significant for each sex (i.e., whether substance use was significantly related to emotional reactivity to arguments). Significant two-way interactions for either male or female adolescents were then further probed as well.


Equation 2: $$L1:{\hat{\text{Emotion}}}_{ijk}=\beta_{0jk}+\beta_{1jk}\left({\text{Argument}}_{ijk}\right)\;\;+\beta_{2jk}\left({\text{Previous}\;{\text{Day}}^{\prime }\text{s}\;\text{Emotion}}_{ijk}\right)\;L2:\beta_{0jk}=\gamma_{00k}+\gamma_{01k}\left({\text{Substance}\;\text{Use}}_{jk}\right)+\gamma_{02k}\left({\text{Age}}_{jk}\right)+u_{0jk}\;\;\beta_{1jk}=\gamma_{10k}+\gamma_{11k}\left({\text{Substance}\;\text{Use}}_{jk}\right)+u_{1jk}\;\;\beta_{2jk}=\gamma_{20k}+u_{2jk}\;\;\text{L}3\;:\gamma_{00k}=\pi_{000}+\pi_{001}\left(\text{Substance}\;{\text{Use}}_{k}\right)+\pi_{002}\left({\text{Age}}_{k}\right)\;\;+\;\pi_{003}(\text{Female})+\pi_{004}(\text{Asian}\;\text{American})\;\;+\;\pi_{005}(\text{European}\;\text{American})\;\;+\;\pi_{006}(\text{Different}\;\text{Ethnic}\;\text{Backgrounds})\;\;+\;\pi_{007}(\text{Parental}\;\text{Education})+r_{00k}\;\;\gamma_{01k}=\pi_{010}+\pi_{011}\left(\text{Female}\right)+r_{01k}\;\gamma_{10k}=\pi_{100}+\pi_{101}\left({\text{Substance}\;\text{Use}}_{k}\right)+\pi_{102}\left(\text{Female}\right)+r_{10k}\;\;\gamma_{11k}=\pi_{110}+\pi_{111}\left(\text{Females}\right)+r_{11k}$$

Analyses in the present study assessed individual differences in emotional reactivity by testing moderation of daily associations, in line with current practices (e.g., Bai et al., 2020; Rahal et al., 2022, 2023; Shirtcliff & Essex, 2008). When considering interaction terms, it is important to recognize that moderated associations tend to be much smaller in magnitude than main effects (e.g., Aguinis et al., 2005; Chaplin, 1991). In a cross-level interaction, the level-2 moderator (substance use) is predicting how the level-1 predictor (arguments) randomly differs across individuals, which can contribute to the small magnitude of the coefficient. Standardized coefficients for cross-level interactions must be contextualized with proportions of variance accounted for by the interaction (e.g., Aguinis et al., 2013). We therefore provide standardized coefficients (0.10 represents a small effect, 0.30 represents a medium effect), as well as two indications of the variance accounted for by a significant cross-level interaction (i.e., the predictor of interest): the $f^{2}$ statistic as an indicator of the degree of variability in the outcome that is accounted for by the interaction term, which is similar to an adjusted $R^{2}$ value in a regression model (0.02 represents a small effect, 0.15 represents a medium effect), and the proportion of variance in the random slope of arguments that is accounted for by substance use in two-way interactions, as well as sex in three-way interactions.

Because we leveraged data from an existing longitudinal dataset, we used sensitivity power analysis to identify the magnitude of two-way interactions that could be detected in the sample and separately in male and female adolescents (Enders et al., 2023). Monte Carlo simulations with 2000 replications and accounting for nesting within waves and participants indicated that models were fully powered (100%) to detect associations with an $f^{2}$ above 0.0075 (Table S2).

Substance use (grand mean-centered), daily stress (wave mean-centered), age (grand mean-centered), sex (dummy-coded, male as reference group), ethnicity (dummy-coded, Latine as reference group [largest ethnic group in sample]), parents’ education (grand mean-centered), and previous day’s emotion were tested as predictors (wave mean-centered). Ethnicity is covaried because Asian youth are less likely to use substances than other groups (Miech et al., 2020).

## 3 | RESULTS

### 3.1 | Descriptive models

Descriptive statistics and correlations between study variables are presented in Table S3. Multilevel models were used to test whether sex, ethnicity, parents’ education, and age related to daily emotion, daily stress, and substance use. Participants reported high levels of positive emotion and low levels of anxious and depressive emotion that did not change with age, *p*s > 0.40. Results indicated that female adolescents were lower in positive emotion, B=-0.15,SE=0.07,p=0.026, 95% Confidence Interval (CI) [−0.28, −0.02], $\beta \;=-0.09,\;f^{2}\;=\;0.02$, and higher in depressive emotion, B=0.09,SE=0.04,p=0.042, 95% CI [0.003, 0.18], $\beta =\;0.06,\;f^{2}\;=\;0.02$, than male adolescents. Adolescents with higher parents’ education reported higher positive emotion, B=0.04,SE=0.02,p=0.031, 95% CI [0.004, 0.08], $\beta \;=\;0.10,\;f^{2}\;=\;0.02$, and higher depressive emotion, B=0.03,SE=0.01,p=0.043, 95% CI [0.0008, 0.05], $\beta \;=\;0.08,\;f^{2}\;=\;0.02$. Adolescents also consistently reported few daily stressors, approximately two per week for each assessment. Daily stressors became less frequent over time, $B\;=-0.03,\;SE\;=\;0.01,\;p<0.001,\;\beta =-0.09,\;f^{2}\;=\;0.06$, and female adolescents reported significantly fewer stressors than male adolescents, B=0.09,SE=0.03,p=0.004, 95% CI [0.03, 0.14], $\beta =\;0.08,\;f^{2}\;=\;0.05$.

**FIGURE 1:**
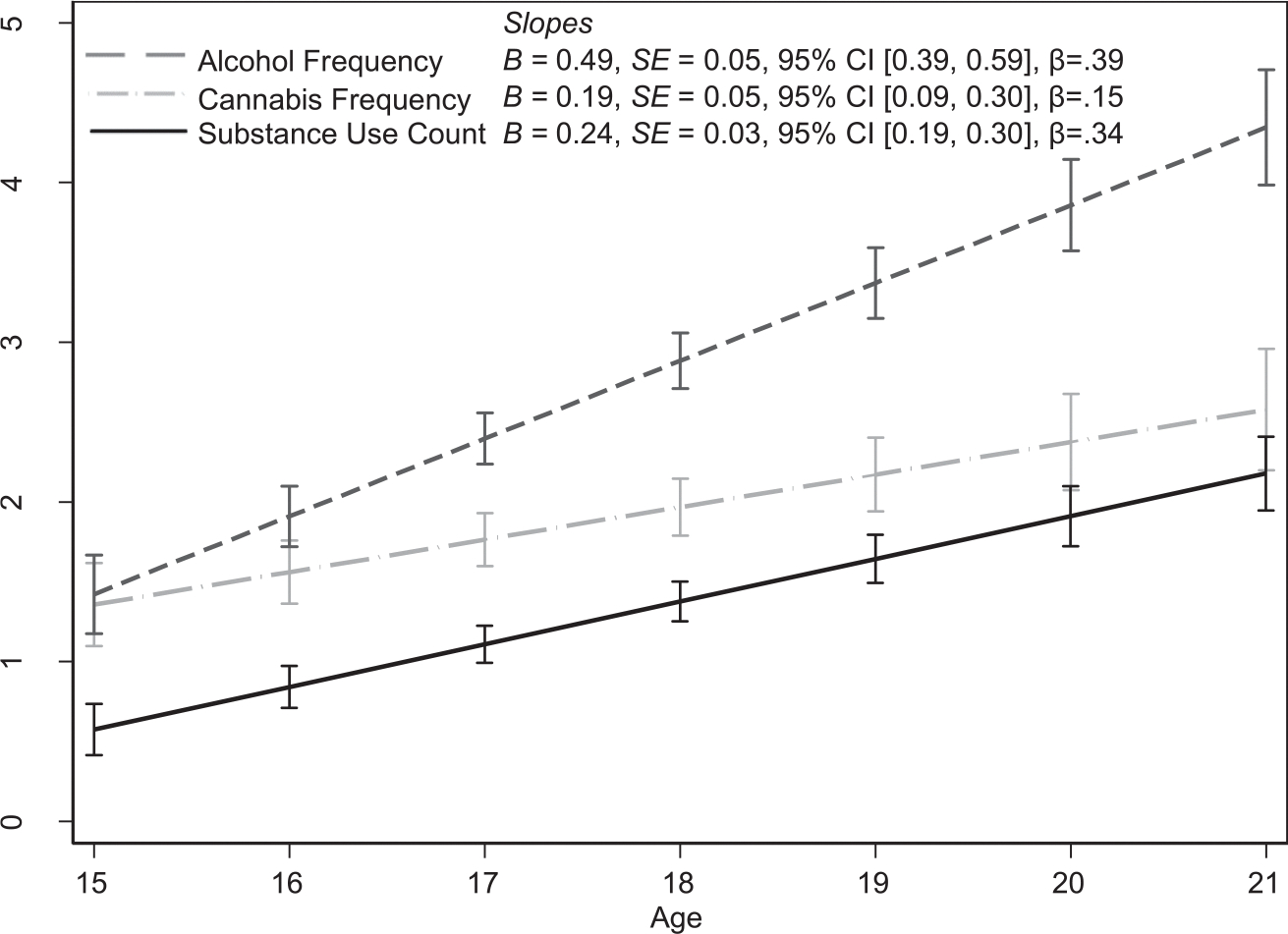
Substance Use as a function of Age. All *p*s < 0.001. Frequency of alcohol and cannabis use over the past year were on a 10-point scale in which 1 represented no use, and lifetime substance use was on a scale of 0–7. CI, confidence interval.

Past year frequency of alcohol and cannabis use and lifetime substance use were low at study entry and increased over time (Figure 1). Female adolescents used cannabis less frequently, B=-0.47,SE=0.20,p=0.021, 95% CI[-0.87,-0.07], $\beta =-0.10,\;f^{2}=\;0.03$, and used fewer substances, B=-0.30,SE=0.13,p=0.020, 95% CI[-0.55,-0.05], $\beta =-0.09,\;f^{2}\;=\;0.02$, than male adolescents. Asian American adolescents reported less frequent alcohol use, B=-0.66,SE=0.22,p=0.002, 95% CI[-1.09,-0.24], $\beta =-0.13,\;f^{2}=\;0.03$; less frequent cannabis use, B=-0.62,SE=0.22,p=0.005, 95% CI[-1.06,-0.18], $\beta =-0.13,\;f^{2}=\;0.04$; using fewer substances, B=-0.43,SE=0.16,p=0.006, 95% CI[-0.74,-0.12], $\beta =-0.12,\;f^{2}=\;0.03$; and fewer daily stressors, B=-0.15,SE=0.04,p<0.001, 95% CI[-0.23,-0.08], $\beta =-0.11,\;f^{2}=\;0.08$, than Latine adolescents.

### 3.2 | Emotional reactivity to stress and substance use

We investigated whether frequency of alcohol and cannabis use over the past year and lifetime substance use (i.e., number of substances ever used by that age) were related to daily emotional reactivity to stress using three-level multilevel models. Models predicting emotion from daily stressors indicated that participants on average reported lower positive emotion and higher anxious and depressive emotion on days when they had more arguments than their average. Therefore, greater emotional reactivity to stress is indicated by a more negative slope for positive emotion and a more positive slope for anxious and depressive emotion.

Models included a Substance Use × Daily Stress cross-level interaction, which tested whether the daily association between stress and each emotion differed by adolescents’ past year frequency of use and lifetime substance use. When interpreting interactions with substance use, an interaction with a negative coefficient would suggest that individuals with greater substance use had a more negative daily slope (i.e., greater positive emotional reactivity to arguments, attenuated negative emotional reactivity to arguments). A positive coefficient would suggest that individuals with greater substance use had a more positive daily slope (i.e., attenuated positive emotional reactivity to arguments, greater negative emotional reactivity to arguments). We also tested whether associations between substance use and daily emotional reactivity to stress differed by adolescent sex by testing a three-way Sex × Substance Use × Daily Stress interaction. We report the percentage of variance in the random slope (i.e., the daily association between arguments and emotion) that is accounted for by substance use for each cross-level interaction.

**FIGURE 2:**
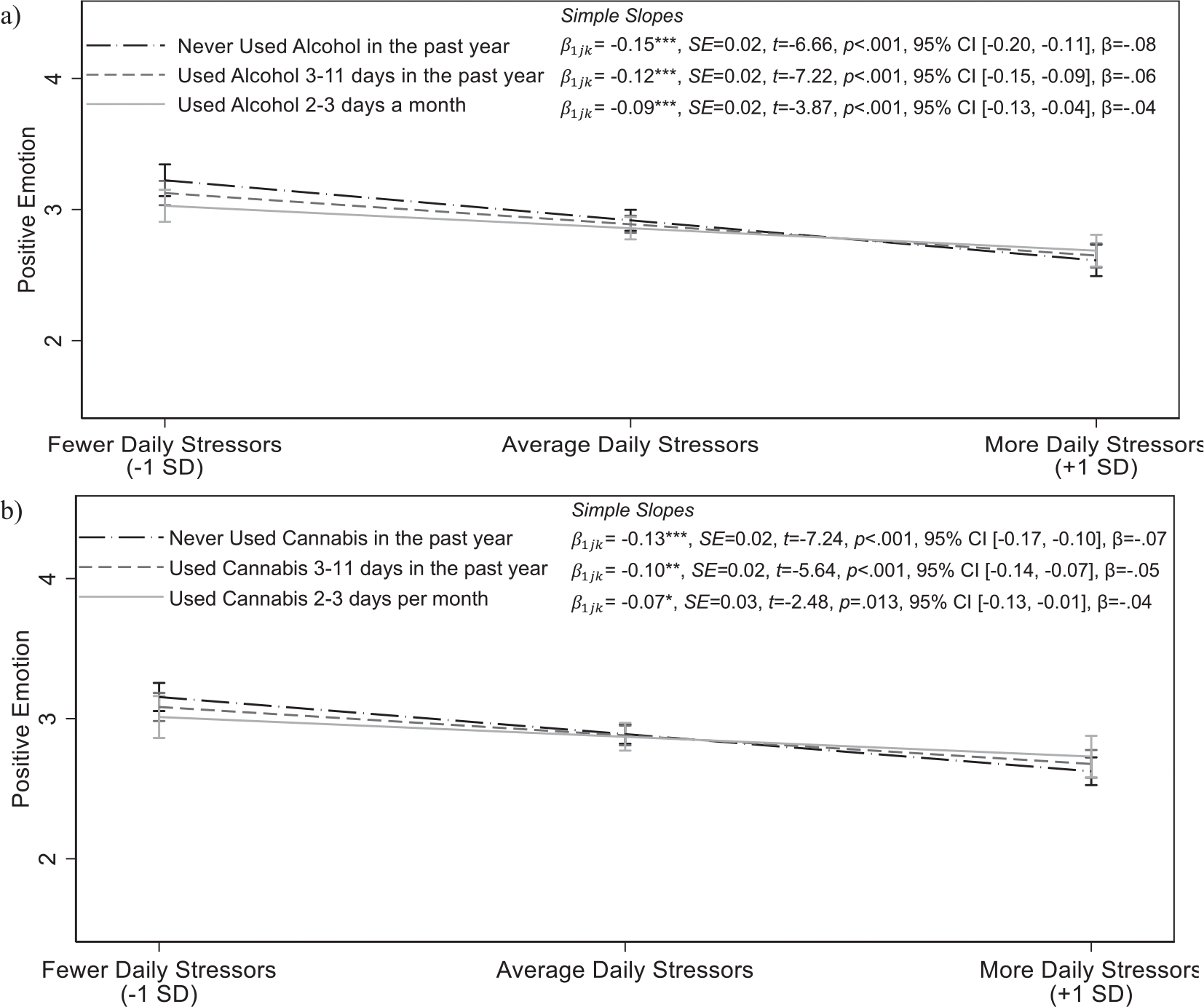
Positive emotion as a function of daily stressors and frequency of alcohol use (a) and frequency of cannabis use (b). Associations controlled for age, ethnicity, parents’ education, and previous day’s emotion. Frequency of alcohol use, and frequency of cannabis use are continuous variables, and associations were probed at approximately one standard deviation below the mean, the mean, and one standard deviation above the mean. Simple slope coefficients are presented next to the legend. Please note that it is common for confidence intervals to overlap despite significantly different slopes (Schenker & Gentleman, 2001). Moderation indicates that the associations between daily arguments and emotion significantly differ, not that the predicted values of emotion are necessarily different at all levels of daily arguments. Participants generally showed a significant degree of emotional reactivity (i.e., lower positive emotion on days when they had more arguments than average), and assessments of regions of significance using the Johnson–Neyman technique revealed that this association was weaker for individuals with higher levels of substance use and no longer significant for individuals who had used alcohol 2 days per week and who had used cannabis one day week. CI, confidence interval; SD, standard deviation. *p < 0.05; **p < 0.01; ***p < 0.001.

Greater frequency of alcohol use, $\gamma_{11k}=0.02,\;SE=0.01,\;p=0.028$, 95% CI [0.002, 0.03], $\beta \;=\;0.02,\;f^{2}\;=\;0.04$, 5.99% random slope variance, and greater cannabis use over the past year $\gamma_{11k}=0.02,\;SE=0.01,\;p=0.049$, 95% CI [0.00003, 0.03], $\beta =\;0.02,\;f^{2}\;=\;0.04$, 6.61% random slope variance, were both related to attenuated positive emotional reactivity to stress (Table S4). Youth who abstained from alcohol and cannabis use over the past year reported the greatest declines in positive emotion on days when they experienced more stressors (i.e., showed the highest degree of positive emotional reactivity to stress; Figure 2). In turn, adolescents who use alcohol and cannabis more frequently over the past year had a smaller decline in positive emotion on days when they experienced more stressors, such that more frequent use was related to attenuated positive emotional reactivity to stress. Importantly, as shown in Figure 2, differences in positive emotion by alcohol frequency were apparent on days when fewer stressors were experienced, as opposed to days when more stressors were experienced. Specifically, youth who used alcohol more frequently over the past year reported lower positive emotion on days when fewer stressors were experienced compared to youth who used substances less frequently, despite comparable levels of positive emotion on days when more stressors were experienced. When the Age × Daily Stress interaction was included in the model, associations between frequency of alcohol use and frequency of marijuana use with positive emotional reactivity to daily stress were no longer significant (*p*s = 0.085 and 0.077, respectively). Lifetime substance use was not related to positive emotional reactivity (i.e., extent of decline in positive emotion) to daily stress, $\gamma_{11k}=0.02,\;SE=0.01,\;p=0.09,\;\beta =\;0.01,\;f^{2}=\;0.04$, 3.67% random slope variance. Associations between substance use measures and positive emotional reactivity to stress did not differ by sex, ps<0.50.

**FIGURE 3:**
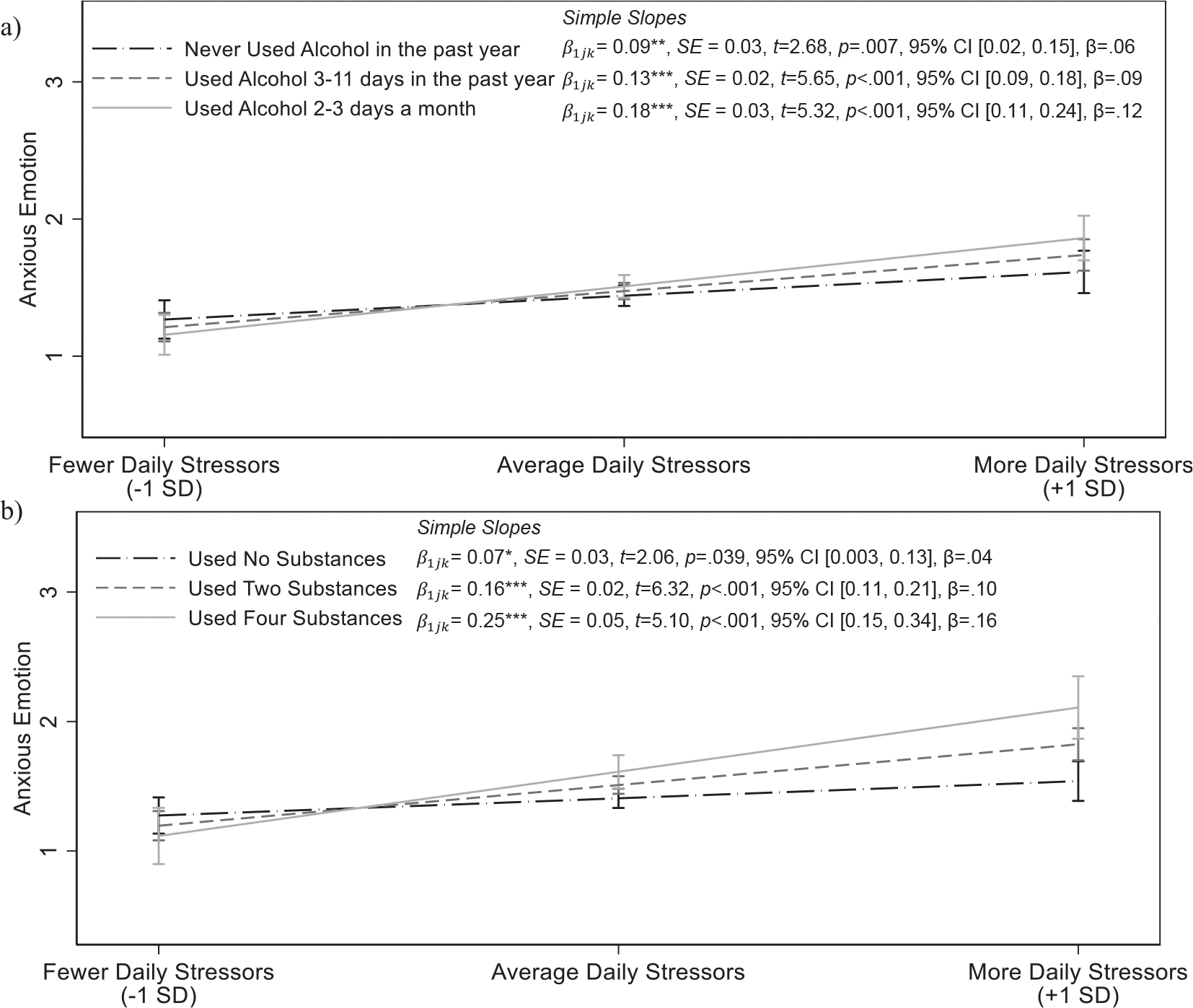
Anxious emotion as a function of daily stressors and frequency of alcohol use (a) and lifetime substance use (b) in female adolescents. Associations controlled for age, ethnicity, parents’ education, and previous day’s emotion. Frequency of alcohol use and lifetime substance use are continuous variables, and associations were probed at values approximately one SD below the mean, the mean, and one SD above the mean. Simple slopes are presented next to the legend. Please note that it is common for confidence intervals to overlap despite significantly different slopes (Schenker & Gentleman, 2001). Moderation indicates that the associations between daily arguments and emotion significantly differ, not that the predicted values of emotion are necessarily different at all levels of daily arguments. Female participants generally showed a significant degree of emotional reactivity (i.e., higher anxious emotion on days when they had more arguments than average), and assessments of regions of significance using the Johnson–Neyman technique revealed that this association significant at all levels of substance use, although stronger associations emerged for female individuals with higher levels of substance use. CI, confidence interval; SD, standard deviation. *p < 0.05; **p < 0.01; ***p < 0.001.

Substance use was related to exaggerated anxious emotional reactivity to stress only among female adolescents (Table S5). The association between frequency of alcohol use over the past year and anxious emotional reactivity to stress significantly differed by sex, $\pi_{111}=0.04,\;SE=0.02,\;p=0.043$, 95% CI [0.001, 0.07], $\beta \;=\;0.02,\;f^{2}=\;0.07$, 1.68% random slope variance. Simple slopes indicated that more frequent alcohol use was associated with exaggerated anxious emotional reactivity to stress in female adolescents, $\gamma_{11k}=0.02,\;SE=0.01,\;p=0.017$, 95% CI [0.003, 0.03], $\beta =\;0.03,\;f^{2}=0.06$, 1.80% random slope variance, but not male adolescents, $\gamma_{11k}=-0.01,\;SE=0.01,\;p=0.4$, 95% CI [−0.03, 0.01], β=-0.02, 0.004% random slope variance (Figure S3a). Female adolescents who used alcohol more frequently showed greater anxious emotion on days when they experienced more daily arguments compared to female adolescents who abstained from alcohol (Figure 3a). Similarly, associations between lifetime use and daily anxious emotional reactivity to stress differed by sex, $\pi_{111}=0.05,\;SE=0.02,\;p=0.040$, 95% CI [0.002, 0.10], $\beta =\;0.02,\;f^{2}=0.08$, 1.77% random slope variance. Female adolescents who used more substances tended to show greater anxious emotion on days when they experienced more arguments, $\gamma_{11k}=0.03,\;SE=0.01,\;p=0.007$, 95% CI [0.008, 0.05], $\beta \;=\;0.04,\;f^{2}=0.05$, 1.50% random slope variance (Figure 3b), and associations were not significant for male adolescents, $\gamma_{11k}=0.00,\;SE=0.01,\;p=0.99$, 95% CI [−0.03, 0.03], β=-0.01, 0.001% random slope variance (Figure S3b). Cannabis use was unrelated to anxious emotional reactivity to stress, p=0.8, and this association did not differ by sex, p=0.3.

**FIGURE 4:**
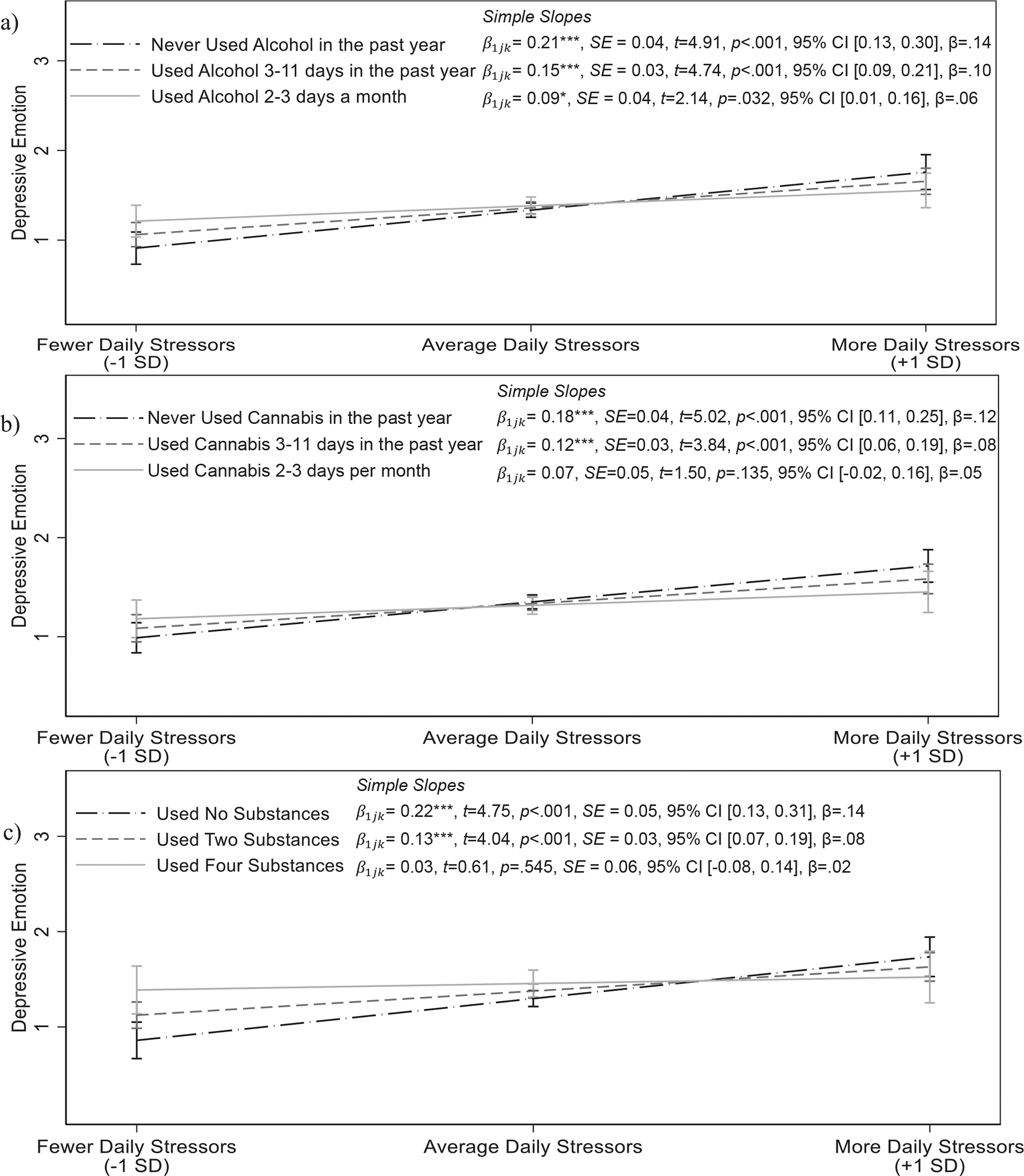
Depressive emotion as a function of daily stressors and frequency of alcohol use (a), frequency of cannabis use (b), and lifetime substance use (c) in male adolescents. Associations controlled for age, ethnicity, parents’ education, and previous day’s emotion. Frequency of alcohol use, frequency of cannabis use, and lifetime substance use are continuous variables, and associations were probed at values approximately one SD below the mean, the mean, and one SD above the mean. Simple slopes are presented next to the legend. Please note that it is common for confidence intervals to overlap despite significantly different slopes (Schenker & Gentleman, 2001). Moderation indicates that the associations between daily arguments and emotion significantly differ, not that the predicted values of emotion are necessarily different at all levels of daily arguments. Male participants showed a significant degree of emotional reactivity (i.e., higher depressive emotion on days when they had more arguments than average), and assessments of regions of significance using the Johnson–Neyman technique revealed that this association was weaker for male individuals with higher levels of substance use and no longer significant for male individuals who had used alcohol once per week or more, who had used cannabis 2–3 days per month or more, and who had used four or more substances. CI, confidence interval; SD, standard deviation. *p < 0.05; **p < 0.01; ***p < 0.001.

Finally, associations between daily substance use and depressive emotional reactivity to stress differed by sex; $\pi_{111}\;=\;0.05,\;SE=0.02,\;p=0.006$, 95% CI [0.01, 0.08], $\beta \;=\;0.03,\;f^{2}\;=\;0.09$, 4.42% random slope variance for frequency of alcohol use; $\pi_{111}\;=\;0.04,\;SE=0.02,\;p=0.026$, 95% CI [0.005, 0.08], $\beta \;=\;0.03,\;f^{2}\;=\;0.10$, 4.40% random slope variance for frequency of cannabis use; $\pi_{111}\;=\;0.08,\;SE=0.03,\;p=0.002$, 95% CI [0.03, 0.13], $\beta \;=\;0.04,\;f^{2}\;=\;0.09$, 5.56% random slope variance for lifetime substance use (Table S6). In contrast to associations for anxious emotion, we found that greater substance use was associated with attenuated depressive emotional reactivity to stress in male adolescents (Figure 4). Although both male and female adolescents generally showed significantly higher depressive emotion on days when they experienced more stressors, male adolescents who used alcohol and cannabis more frequently over the past year and who used more substances also reported smaller increases in depressive emotion on days when they experienced more daily stress; $\gamma_{11k}\;=-0.03,\;SE=0.01,\;p=0.001$, 95% CI [−0.04, −0.01], $\beta \;=-0.04,\;f^{2}\;=\;0.09$, 5.43% random slope variance for frequency of alcohol use; $\gamma_{11k}\;=-0.03,\;SE=0.01,\;p=0.001$, 95% CI [−0.04, −0.01], $\beta =-0.04,\;f^{2}=\;0.08$, 6.10% random slope variance for frequency of cannabis use, $\gamma_{11k}\;=-0.05,\;SE=0.01,\;p<0.001$, 95% CI [−0.08, −0.02], $\beta =-0.04,\;f^{2}=\;0.12$, 5.59% random slope variance for lifetime substance use. Among female adolescents, more frequent past year alcohol use and greater lifetime use were associated with marginally greater depressive emotional reactivity to stress in line with associations for anxious emotional reactivity to stress. Female adolescents who used alcohol more frequently, $\gamma_{11k}\;=\;0.01,\;SE=0.01,\;p=0.094$, 95% CI [−0.002, 0.03], β=0.02, 1.28% random slope variance, and used more substances, $\gamma_{11k}\;=\;0.02,\;SE=0.01,\;p=0.060$, 95% CI [−0.0008, 0.04], β=0.03, 1.78% random slope variance, showed marginally greater increases in depressive emotion on days when they experienced more arguments (Figure S4). Frequency of cannabis use over the past year was not associated with daily depressive emotional reactivity to arguments for female adolescents, $\gamma_{11k}\;=\;0.00,\;SE=0.01,\;p=0.620$, 95% CI [−0.01, 0.02], β=0.02, 0.001% random slope variance.

Significant associations were tested again controlling for emotional variability (calculated as the individual standard deviation across days) and mean number of daily stressors (wave mean-centered) to determine whether associations between substance use and emotional reactivity to stress were unique from associations with more general fluctuations in emotion and frequency of daily stressors. These models also covaried for whether participants were living with their parents (94% at Wave 2, 82% at Wave 3) or had a romantic partner (27% at Wave 2, 42% at Wave 3) at each wave (0 = no, 1 = yes) to account for opportunity for arguments. Follow-up analyses resulted in a nearly identical pattern of results. All results are summarized in Table S7.

## 4 | DISCUSSION

Guided by a developmental psychopathology framework (Cicchetti & Cohen, 1995; Cichetti & Rogosch, 2002), we assessed how frequency of alcohol and cannabis use in adolescence is related to individuals’ emotional reactivity to daily stressors. Results revealed that associations between substance use and emotional reactivity to daily stress varied by emotional valence and sex. More frequent use of alcohol and cannabis were related to attenuated positive emotional reactivity to stress among male and female adolescents. When examining two dimensions of negative emotional reactivity (i.e., anxious and depressive emotion), we found that more frequent alcohol use over the past year and greater lifetime substance use were associated with exaggerated anxious emotional reactivity to stress in female adolescents, whereas more frequent use of alcohol and cannabis over the past year and greater lifetime substance use were associated with attenuated depressive emotional reactivity to stress in male adolescents. Associations for the substance use measure of primary interest, frequency of alcohol use, were consistently replicated for either frequency of cannabis use (for positive and depressive emotional reactivity) or lifetime substance use (for anxious and depressive emotional reactivity). Results suggest that substance use in adolescence may be related to psychopathology including depressive symptoms in adulthood because these youth are also showing differences in daily emotional reactivity to stress. Study results extend past findings relating acute emotional reactivity to substance use risk by identifying concurrent associations between substance use and daily emotional reactivity to stress, suggesting that substance use may be tied to adolescents’ daily experiences and thereby incur risk. It is possible that use of substances can impact how youth respond to subsequent daily experiences, which may also contribute to greater substance use over time, in addition to poorer social relationships and mental health.

### 4.1 | Substance use and emotional reactivity to stress

From a developmental psychopathology framework (Cicchetti & Cohen, 1995; Cichetti & Rogosch, 2002), findings suggest that substance use is related to daily emotion processes, which can be a potential microsystem pathway relating substance use to psychopathology risk and other outcomes. Repeated use of substances could impact an adolescent’s daily experiences including their emotion regulation and capacity to respond to stress (Weiss et al., 2017). Psychopathology could also be a mediating pathway, as mental health problems that are often comorbid with substance use (e.g., greater internalizing and externalizing problems, respectively) may similarly relate to exaggerated and attenuated emotional reactivity to stress through mutually reinforcing pathways (Danzo et al., 2021; Felton et al., 2015).

More frequent substance use was associated with attenuated positive emotional reactivity to stress for both male and female youth. Statistical models also indicated that youth who used substances more frequently showed consistently lower positive emotion, irrespective of daily stressors, whereas youth who used substances less frequently tended to report higher positive emotion on days when they experienced relatively fewer stressors. Results extend prior findings that have largely emphasized high negative rather than low positive emotion as a risk factor for substance use (Rusby et al., 2019; Shadur et al., 2015). Participants on average did not report the scale minimum of positive emotion, suggesting that youth who used substances more frequently had both chronically lower positive emotion and low positive emotional reactivity to daily stress.

It is possible that substance use was related to attenuated positive emotional reactivity because youth who are not stimulated by daily experiences may be motivated to use substances to enhance positive emotion (Zuckerman, 2007). Frequent substance use could also disrupt adolescents’ capacity for self-regulation, impact well-being and daily experiences, and consequently shape their positive emotional reactivity to stress (Parrish et al., 2016; Weiss et al., 2017), which may explain associations with frequency but not lifetime substance use. Associations also may not have emerged for lifetime substance use because youth who used more substances may have experimented with substances and then discontinued use. Although statistical models predicted emotion from substance use, associations are likely bidirectional. Difficulties with emotion regulation and heightened chronic negative emotion could position adolescents to be exposed to substances through deviant peers and more motivated to use substances to cope with stress (e.g., Brook et al., 2011; Fox et al., 2011; Gallegos et al., 2021).

### 4.2 | Sex differences in associations between substance use and emotional reactivity to stress

Sex differences emerged in associations between substance use and anxious and depressive emotional reactivity to stress, in agreement with sex-specific associations from other studies (e.g., Chaplin et al., 2019). More frequent use of alcohol over the past year and greater lifetime use were associated with exaggerated anxious emotional reactivity among female adolescents. This result aligns with prior findings that exaggerated emotional variability and emotional reactivity incur risk for poor mental health including depressive symptoms (Bai et al., 2020; Myin-Germeys et al., 2003). A somewhat consistent pattern of results—with more frequent alcohol use over the past year and greater lifetime substance use relating to exaggerated emotional reactivity to stress in female adolescents—was marginally significant for depressive emotion as well. Sex differences may have emerged because greater reactivity to interpersonal stress can result in internalizing problems, which are more prevalent and more related to substance use in female than in male adolescents (e.g., Danzo et al., 2021). Greater stress and conflict also tend to be more strongly related to substance use disorders among female than male adolescents (Skeer et al., 2011). Cannabis use may not relate to anxious emotional reactivity to stress because it relates to differences in other aspects of anxious emotion, irrespective of daily stress. For instance, more frequent cannabis use has been related to greater anxious mood lability and anxiety in adolescence and young adulthood (Epstein et al., 2015; Rusby et al., 2019). Cannabis use was also low in this sample, limiting ability to detect associations.

In turn, greater past year frequency of use and lifetime substance use were related to dampened depressive emotional reactivity to stress in male adolescents. These results expand prior research that has related attenuated emotional flexibility to depression and psychopathy (e.g., Truedsson et al., 2019). Attenuated depressive emotional reactivity to stress may indicate lower sensitivity to stress, and under-arousal from daily experiences could motivate later substance use (Zuckerman, 2007). Attenuated emotional reactivity to positive and negative images has been associated with greater psychopathology including externalizing problems and callous-unemotional traits, both of which have been related to greater substance use and tend to be higher in male than in female adolescents (Hillege et al., 2010; Truedsson et al., 2019).

Differences in emotion socialization and motives for substance use have also been theorized to contribute to sex differences in pathways to substance use (e.g., Chaplin et al., 2018). Male adolescents are often more socialized to avoid expressing their emotions than female adolescents (Fivush et al., 2000). Therefore, difficulties with stress regulation may be indexed by attenuated reactivity to stress in male adolescents and exaggerated reactivity in female adolescents. Motives also differ by sex such that male adolescents are more likely to report motives to use substances to promote sociability and to enhance arousal, whereas female adolescents are more likely to use substances to reduce distress (Kuntsche et al., 2015). It may be that youth who use substances to promote arousal may also be less influenced by social stressors and consequently show attenuated emotional reactivity to stress. In turn, youth who are motivated by beliefs that substances can reduce distress may also be more sensitive, and thus responsive, to stress. Future studies will need to investigate whether these factors account for sex-based differences in associations.

### 4.3 | Study implications

Associations were small in magnitude, highlighting that substance use is related to emotional reactivity as one distinct aspect of emotion, over and above overall levels of emotion, and that many factors beyond substance use and arguments relate to daily positive and negative emotion. Therefore emotional reactivity to stress could be targeted as an avenue for intervention, but it would likely be targeted in conjunction with other pathways. The modest effect sizes suggest that it is possible that substance use and emotional reactivity may relate to one another through another variable, such as psychopathology. Study findings may have implications for sex-specific substance use treatment and for identifying youth at early risk of substance use. Substance use may incur consequences by altering adolescents’ responses to stress, and substance use treatment programs for youth may also benefit from incorporating trainings regarding coping with stress, including aspects of mindfulness and acceptance (Broderick, 2013). Emerging evidence also suggests that cultivating a sense of purpose can promote emotion regulatory skills, dampen emotional reactivity to positive and negative events, and reduce risk of substance use (Hill et al., 2022; Minehan et al., 2000), suggesting that interventions, activities, or discussions that cultivate purpose might reduce substance use and emotional reactivity to stress.

Family- and school-based interventions can incorporate discussions regarding how to respond to stress and openly address how substance use can be a tempting but counterproductive means of responding to stress, acknowledging potential differences in how male and female youth may be inclined to respond to stress. Parents can observe and track their children’s general emotional reactivity to daily stressors. Interventions may also alter emotional reactivity to stress among at-risk youth by incorporating positive parenting practices that have been previously associated with differences in negative emotional reactivity to daily stressors, including higher parental warmth and better communication (e.g., Lippold et al., 2016).

### 4.4 | Limitations

The study was limited by the measurement of daily emotions and substance use. Findings could be strengthened by measuring discrete emotions (e.g., anger) and emotional arousal. We used an extensively used and well-validated method for assessing daily linkages between stressors and emotion (Almeida, 2005; Herres et al., 2016), although it is possible that external factors could influence emotion reported at the end of the day or that emotion could contribute to interpersonal stress. The daily protocol was technically administered shortly after reports of substance use, although daily protocols typically capture general patterns of individuals’ daily experiences (Shiffman, 2009), and we would not expect estimates of emotional reactivity from checklists to greatly deviate across the span of weeks or months. Although the present study assessed argument frequency, arguments can vary in intensity. Future studies can account for how intense an argument was using subjective ratings of intensity or perceived stressfulness.

There were also limitations to the accelerated longitudinal design of the study. There was attrition across the study, and youth of lower parental education and Asian American backgrounds had higher attrition compared to other participants. The 2-year interval between assessments precluded rigorous examination of directionality of associations, and future longitudinal studies with more frequent assessments (e.g., yearly) can examine prospective and bidirectional associations between emotional reactivity to stress and substance use. Finally, participants’ first reports were collected between 2011 and 2012, and vaping has since become more prevalent and should be measured in future studies. The COVID-19 pandemic also influenced both adolescents’ substance use and emotion, such that associations between emotional reactivity to stress and substance use may have shifted during this unique period.

## 5 | CONCLUSION

Although emotion and stress are related to substance use, limited research has examined whether substance use relates to emotional reactivity to daily stress during adolescence, a period when youth show increasing substance use (Miech et al., 2020). The present study found that greater substance use, with respect to frequency of alcohol and cannabis use over the past year and lifetime substance use, were related to emotional reactivity to stress among adolescents, over and above emotional variability, with associations differing by sex and emotional valence. More frequent use of alcohol and cannabis was related to attenuated positive emotional reactivity to daily stress among male and female adolescents. More frequent alcohol use over the past year and greater lifetime use were related to exaggerated anxious emotional reactivity to stress among female adolescents, whereas greater substance use frequency and lifetime use were related to attenuated depressive emotional reactivity to stress among male adolescents. These findings support conceptual models that relate emotional reactivity to stress to substance use differentially by sex (Chaplin et al., 2018). Further research is needed to identify how emotional reactivity to stress relates to substance use, the temporality of associations, and whether differences in emotion socialization or substance use motives may explain sex-specific patterns of associations.

## Supplementary Material

supplement

SUPPORTING INFORMATION

Additional supporting information can be found online in the Supporting Information section at the end of this article.

## Data Availability

Data were collected as part of a longitudinal study. Data are available upon request. Code and materials are provided at https://osf.io/53kry.
