# Supplementary material for: Associations between emotional reactivity to stress and adolescent substance use: Differences by sex and valence
Source: Stress Health. Author manuscript; Available in PMC 2025 Dec 10. (PMC12691974; doi:10.1002/smi.3420)
Supplement: supplement [file NIHMS2119153-supplement-supplement.docx]

**Supplemental Information for Associations between Emotional Reactivity to Stress and Adolescent Substance Use: Differences by Sex and Valence**

Table S1 presents inter-item reliability values for daily emotion for the full sample and disaggregated by sex and ethnicity. Table S2 presents the results of sensitivity power analyses for detecting two-way interactions between substance use and daily stress as predictors of emotion in male participants, female participants, and the full sample. Tables S3 presents descriptive statistics and correlations for study variables at each of the three data collection assessments. Tables S4-S6 present tabulated results for primary analyses, for which three-level multilevel models tested associations between frequency of alcohol use over the past year, cannabis use over the past year, and lifetime substance use and daily emotional reactivity. Table S7 summarizes study results by sex. Figures S1 provides a study schematic. Figure S2 visually depicts the conceptual model guiding the research questions for this study. Figure S3 presents associations for anxious emotion as a function of daily stressors and frequency of alcohol use over the past year, frequency of cannabis use over the past year, and lifetime substance use among male adolescents (figure for female adolescents presented in the main-text). Figure S4 presents associations for depressive emotion as a function of daily stressors and frequency of alcohol use over the past year, frequency of cannabis use over the past year, and lifetime substance use among female adolescents (figure for male adolescents presented in the main-text). Daily measures of stressors and emotion and wave measures of substance use are provided. Syntax for all models in Stata 16.1 are also provided.

*Table S1*. Alpha reliabilities for daily emotion items by sex and ethnic background.

| Sample | Assessment | Positive Emotion | Anxious Emotion | Depressive Emotion |
| --- | --- | --- | --- | --- |
| Full Sample | 1 | .89 | .80 | .74 |
|  | 2 | .89 | .80 | .77 |
|  | 3 | .90 | .82 | .78 |
| Female | 1 | .87 | .80 | .73 |
|  | 2 | .89 | .81 | .77 |
|  | 3 | .88 | .84 | .77 |
| Male | 1 | .91 | .81 | .76 |
|  | 2 | .89 | .78 | .77 |
|  | 3 | .90 | .75 | .79 |
| Latine | 1 | .88 | .80 | .74 |
|  | 2 | .87 | .78 | .76 |
|  | 3 | .89 | .81 | .80 |
| Asian American | 1 | .89 | .76 | .69 |
|  | 2 | .90 | .82 | .83 |
|  | 3 | .90 | .77 | .82 |
| European American | 1 | .90 | .81 | .75 |
|  | 2 | .92 | .82 | .76 |
|  | 3 | .90 | .81 | .73 |
| Different Ethnic Background | 1 | .89 | .89 | .82 |
|  | 2 | .89 | .78 | .75 |
|  | 3 | .89 | .90 | .77 |

*Table S2*. Detectable magnitude of two-way interactions (*f*^2^) for male participants (*n*=144), female participants (*n*=186), and the full sample (*N*=330) based on sensitivity power analyses using Monte Carlo simulations with 2000 replications that accounted for nesting of days within individuals.

|  | 80% | 100% |
| --- | --- | --- |
| Male Participants | .0035 | .0074 |
| Female Participants | .0025 | .0053 |
| All Participants | .0017 | .0033 |

Note: Values were calculated as *R*^2^_product_ values and converted to *f*^2^ metrics of effect size using the following formula: *f*^2^=$\frac{R^{2}}{1-R^{2}}$. Values were calculated using the mlmpower package in R (Enders et al., 2023).

*Table S3*. Descriptive statistics and correlations for study variables at the first wave of data collection.

| Wave | Variable | *M* | *SD* | *Min* | *Max* | 1. | 2. | 3. | 4. | 5. | 6. | 7. |
| --- | --- | --- | --- | --- | --- | --- | --- | --- | --- | --- | --- | --- |
| Wave 1  (10^th^ &11^th^ Grade) | 1. Positive Emotion | 2.88 | 0.71 | 1.12 | 4.91 | — |  |  |  |  |  |  |
|  | 2. Depressive Emotion | 1.41 | 0.46 | 1.00 | 4.00 | -.09 | — |  |  |  |  |  |
|  | 3. Anxious Emotion | 1.44 | 0.52 | 1.00 | 4.15 | -.02 | .76*** | — |  |  |  |  |
|  | 4. Daily Stressors | 0.30 | 0.36 | 0.00 | 2.00 | -.03 | .35*** | .38*** | — |  |  |  |
|  | 5. Alcohol Frequency | 2.11 | 1.64 | 1.00 | 10.00 | -.10 | .11 | .10 | .20*** | — |  |  |
|  | 6. Cannabis Frequency | 1.64 | 1.68 | 1.00 | 9.00 | .04 | .03 | .05 | .10 | .54*** | — |  |
|  | 7. Lifetime Substance Use | 0.96 | 1.19 | 0.00 | 5.00 | -.09 | .11 | .15** | .18** | .68*** | .63*** | — |
| Wave 2   (12^th^ Grade & 1-Year  Post-High School) | 1. Positive Emotion | 2.94 | 0.67 | 1.34 | 4.76 | — |  |  |  |  |  |  |
|  | 2. Depressive Emotion | 1.43 | 0.47 | 1.00 | 3.82 | -.19** | — |  |  |  |  |  |
|  | 3. Anxious Emotion | 1.44 | 0.47 | 1.00 | 3.27 | -.06 | .71*** | — |  |  |  |  |
|  | 4. Daily Stressors | 0.26 | 0.28 | 0.00 | 1.67 | -.14 | .21** | .27*** | — |  |  |  |
|  | 5. Alcohol Frequency | 2.98 | 2.05 | 1.00 | 10.00 | -.16* | -.01 | -.01 | .19** | — |  |  |
|  | 6. Cannabis Frequency | 2.02 | 2.07 | 1.00 | 10.00 | -.14 | -.05 | -.03 | .10 | .49*** | — |  |
|  | 7. Lifetime Substance Use | 1.42 | 1.38 | 0.00 | 6.00 | -.13 | .09 | .06 | .17* | .71*** | .58*** | — |
| Wave 3  (2-3 Years  Post-High School) | 1. Positive Emotion | 2.96 | 0.67 | 1.61 | 4.78 | — |  |  |  |  |  |  |
|  | 2. Depressive Emotion | 1.44 | 0.52 | 1.00 | 3.40 | -.16* | — |  |  |  |  |  |
|  | 3. Anxious Emotion | 1.46 | 0.50 | 1.00 | 3.25 | -.20* | .82*** | — |  |  |  |  |
|  | 4. Daily Stressors | 0.18 | 0.23 | 0.00 | 1.33 | -.13 | .18* | .20* | — |  |  |  |
|  | 5. Alcohol Frequency | 4.03 | 2.30 | 1.00 | 10.00 | .10 | .08 | .04 | .17* | — |  |  |
|  | 6. Cannabis Frequency | 2.54 | 2.49 | 1.00 | 10.00 | .02 | -.01 | -.05 | -.04 | .49*** | — |  |
|  | 7. Lifetime Substance Use | 2.07 | 1.64 | 0.00 | 7.00 | -.06 | .13 | .12 | .10 | .66*** | .56*** | — |

*Note*: **p*<.05, ***p*<.01, ****p*<.001. Values for emotion and stressors were averaged across the 15 days.

*Table S4.* Positive emotion as a function of daily stressors and frequency of alcohol use (column 1), frequency of cannabis use (column 2), and lifetime substance use (column 3).

|  |  | Frequency of Alcohol Use | | | Frequency of Cannabis Use | | | Lifetime Substance Use | | |
| --- | --- | --- | --- | --- | --- | --- | --- | --- | --- | --- |
| *Fixed Effects* |  | *B* | *SE* | *β* | *B* | *SE* | *β* | *B* | *SE* | *β* |
| Intercept |  | 2.97*** | 0.06 | .01 | 2.97*** | 0.06 | .01 | 2.98*** | 0.06 | .01 |
| Daily Stressors | $\beta_{1jk}$ | -0.12*** | 0.02 | -.06 | -0.12*** | 0.02 | -.06 | -0.12*** | 0.02 | -.06 |
| Substance Use | $\gamma_{01k}$ | -0.02 | 0.01 | -.04 | -0.01 | 0.01 | -.01 | -0.03 | 0.02 | -.05 |
| Daily Stressors × Substance Use | $\gamma_{11k}$ | 0.02* | 0.01 | .02 | 0.02* | 0.01 | .02 | 0.02 | 0.01 | .01 |
| Female | $\pi_{003}$ | -0.17* | 0.07 | -.09 | -0.17* | 0.07 | -.09 | -0.17** | 0.07 | -.10 |
| Asian American | $\pi_{004}$ | -0.09 | 0.09 | -.04 | -0.08 | 0.09 | -.03 | -0.09 | 0.09 | -.04 |
| European American | $\pi_{005}$ | 0.12 | 0.08 | .06 | 0.10 | 0.08 | .06 | 0.12 | 0.08 | .06 |
| Different Ethnic Backgrounds | $\pi_{006}$ | -0.09 | 0.15 | -.02 | -0.09 | 0.15 | -.02 | -0.10 | 0.15 | -.03 |
| Parental Education | $\pi_{007}$ | 0.05* | 0.02 | .10 | 0.05* | 0.02 | .09 | 0.05* | 0.02 | .10 |
| Age | $\gamma_{02k}$ | 0.04** | 0.01 | .07 | 0.03* | 0.01 | .06 | 0.04** | 0.01 | .07 |
| Previous Day’s Emotion | $\beta_{2jk}$ | 0.22*** | 0.01 | .14 | 0.22*** | 0.01 | .14 | 0.22*** | 0.01 | .14 |
| *Random Effects* | | Variance | *SE* |  | Variance | *SE* |  | Variance | *SE* |  |
| *Participant* | |  |  |  |  |  |  |  |  |  |
| Substance Use | | 0.00 | 0.00 |  | 0.00 | 0.00 |  | 0.00 | 0.00 |  |
| Age | | 0.00 | 0.00 |  | 0.01 | 0.00 |  | 0.00 | 0.00 |  |
| Participant Intercept | | 0.25*** | 0.03 |  | 0.25*** | 0.03 |  | 0.25*** | 0.03 |  |
| *Wave* | |  |  |  |  |  |  |  |  |  |
| Arguments | | 0.04*** | 0.01 |  | 0.03*** | 0.01 |  | 0.03*** | 0.01 |  |
| Previous Day's Emotion | | 0.02*** | 0.00 |  | 0.02*** | 0.00 |  | 0.02*** | 0.00 |  |
| Wave Intercept | | 0.16*** | 0.02 |  | 0.16*** | 0.02 |  | 0.16*** | 0.02 |  |
| Arguments* Previous Day's Emotion | | 0.01* | 0.00 |  | 0.01* | 0.00 |  | 0.01* | 0.00 |  |
| Arguments*Wave Intercept | | -0.01 | 0.01 |  | -0.01 | 0.01 |  | -0.01 | 0.01 |  |
| Previous Day's Emotion*Wave Intercept | | -0.02** | 0.01 |  | -0.02** | 0.01 |  | -0.02** | 0.01 |  |
| Residual | | 0.27*** | 0.00 |  | 0.27*** | 0.00 |  | 0.27*** | 0.00 |  |

*Note*: Daily stressors were centered at the wave mean, substance use was centered at the grand mean, female was dummy-coded (male as reference group), ethnicity was dummy-coded (Latine as reference group), parents’ education was centered at the grand mean, age was centered at the grand mean, and previous day’s emotion was centered at the wave mean. **p*<.05, ***p*<.01, ****p*<.001.

*Table S5.* Anxious emotion as a function of daily stressors and frequency of alcohol use (column 1), frequency of cannabis use (column 2), and lifetime substance use (column 3).

|  | |  | | Frequency of Alcohol Use | | | Frequency of Cannabis Use | | | Lifetime Substance Use | | | |
| --- | --- | --- | --- | --- | --- | --- | --- | --- | --- | --- | --- | --- | --- |
| *Fixed Effects* |  | | *B* | | *SE* | *β* | *B* | *SE* | *β* | *B* | *SE* | *β* |  |
| Intercept |  | | 1.40*** | | 0.04 | .01 | 1.39*** | 0.04 | .01 | 1.39*** | 0.04 | .01 |  |
| Daily Stressors | $\beta_{1jk}$ | | 0.16*** | | 0.03 | .09 | 0.16*** | 0.03 | .09 | 0.16*** | 0.03 | .09 |  |
| Substance Use | $\gamma_{01k}$ | | 0.01 | | 0.01 | .04 | 0.00 | 0.01 | .00 | 0.04 | 0.02 | .09 |  |
| Daily Stressors × Substance Use | $\gamma_{11k}$ | | -0.01 | | 0.01 | .01 | -0.01 | 0.01 | .01 | -0.01 | 0.02 | .02 |  |
| Female | $\pi_{003}$ | | 0.09 | | 0.05 | .06 | 0.10* | 0.05 | .07 | 0.10* | 0.05 | .07 |  |
| Daily Stressors × Female | $\pi_{102}$ | | -0.03 | | 0.04 | -.01 | -0.03 | 0.04 | -.01 | -0.03 | 0.04 | -.01 |  |
| Substance Use × Female | $\pi_{011}$ | | 0.01 | | 0.02 | .01 | 0.00 | 0.02 | .00 | 0.01 | 0.03 | .01 |  |
| Daily Stressors × Substance Use × Female | $\pi_{111}$ | | 0.04* | | 0.02 | .02 | 0.02 | 0.02 | .01 | 0.05* | 0.02 | .02 |  |
| Asian American | $\pi_{004}$ | | -0.06 | | 0.06 | -.04 | -0.07 | 0.06 | -.04 | -0.05 | 0.06 | -.03 |  |
| European American | $\pi_{005}$ | | -0.06 | | 0.06 | -.04 | -0.05 | 0.06 | -.04 | -0.05 | 0.06 | -.03 |  |
| Different Ethnic Backgrounds | $\pi_{006}$ | | 0.24* | | 0.10 | .08 | 0.24* | 0.10 | .08 | 0.24* | 0.10 | .08 |  |
| Parental Education | $\pi_{007}$ | | 0.02 | | 0.01 | .05 | 0.02 | 0.01 | .05 | 0.02 | 0.01 | .05 |  |
| Age | $\gamma_{02k}$ | | 0.00 | | 0.01 | .00 | 0.00 | 0.01 | .01 | -0.01 | 0.01 | -.01 |  |
| Previous Day’s Emotion | $\beta_{2jk}$ | | 0.11*** | | 0.02 | .08 | 0.11*** | 0.02 | .08 | 0.11*** | 0.02 | .08 |  |
| *Random Effects* | | | Variance | | *SE* |  | Variance | *SE* |  | Variance | *SE* |  |  |
| *Participant* | | |  | |  |  |  |  |  |  |  |  |  |
| Substance Use | | | 0.00 | | 0.00 |  | 0.00 | 0.00 |  | 0.00 | 0.00 |  |  |
| Age | | | 0.00 | | 0.00 |  | 0.00 | 0.00 |  | 0.00 | 0.00 |  |  |
| Participant Intercept | | | 0.09*** | | 0.02 |  | 0.09*** | 0.01 |  | 0.09*** | 0.01 |  |  |
| *Wave* | | |  | |  |  |  |  |  |  |  |  |  |
| Arguments | | | 0.08*** | | 0.01 |  | 0.08*** | 0.01 |  | 0.08*** | 0.01 |  |  |
| Previous Day's Emotion | | | 0.04*** | | 0.01 |  | 0.04*** | 0.01 |  | 0.04*** | 0.01 |  |  |
| Wave Intercept | | | 0.13*** | | 0.02 |  | 0.13*** | 0.02 |  | 0.12*** | 0.02 |  |  |
| Arguments* Previous Day's Emotion | | | 0.00 | | 0.01 |  | 0.00 | 0.01 |  | 0.00 | 0.01 |  |  |
| Arguments*Wave Intercept | | | 0.03** | | 0.01 |  | 0.03** | 0.01 |  | 0.02** | 0.01 |  |  |
| Previous Day's Emotion*Wave Intercept | | | 0.02*** | | 0.01 |  | 0.02*** | 0.01 |  | 0.02*** | 0.01 |  |  |
| Residual | | | 0.21*** | | 0.00 |  | 0.21*** | 0.00 |  | 0.21*** | 0.00 |  |  |

*Note*: Daily stressors were centered at the wave mean, substance use was centered at the grand mean, female was dummy-coded (male as reference group), ethnicity was dummy-coded (Latine as reference group), parents’ education was centered at the grand mean, age was centered at the grand mean, and previous day’s emotion was centered at the wave mean. **p*<.05, ***p*<.01, ****p*<.001.

*Table S6.* Depressive emotion as a function of daily stressors and frequency of alcohol use (column 1), frequency of cannabis use (column 2), and lifetime substance use (column 3).

|  | |  | | Frequency of Alcohol Use | | | Frequency of Cannabis Use | | | Lifetime Substance Use | | | |
| --- | --- | --- | --- | --- | --- | --- | --- | --- | --- | --- | --- | --- | --- |
| *Fixed Effects* |  | | *B* | | *SE* | *β* | *B* | *SE* | *β* | *B* | *SE* | *β* |  |
| Intercept |  | | 1.35*** | | 0.04 | .00 | 1.34*** | 0.04 | .01 | 1.34*** | 0.04 | .00 |  |
| Daily Stressors | $\beta_{1jk}$ | | 0.15*** | | 0.03 | .12 | 0.15*** | 0.03 | .12 | 0.16*** | 0.03 | .12 |  |
| Substance Use | $\gamma_{01k}$ | | 0.01 | | 0.01 | .06 | -0.01 | 0.01 | .02 | 0.04 | 0.02 | .07 |  |
| Daily Stressors × Substance Use | $\gamma_{11k}$ | | -0.03* | | 0.01 | .00 | -0.03* | 0.01 | .00 | -0.05* | 0.02 | .00 |  |
| Female | $\pi_{003}$ | | 0.09* | | 0.04 | .06 | 0.10* | 0.04 | .08 | 0.10* | 0.04 | .07 |  |
| Daily Stressors × Female | $\pi_{102}$ | | 0.06 | | 0.04 | .02 | 0.06 | 0.04 | .02 | 0.05 | 0.04 | .02 |  |
| Substance Use × Female | $\pi_{011}$ | | 0.01 | | 0.02 | .02 | 0.03 | 0.02 | .04 | -0.01 | 0.03 | -.01 |  |
| Daily Stressors × Substance Use × Female | $\pi_{111}$ | | 0.05** | | 0.02 | .03 | 0.04* | 0.02 | .03 | 0.08** | 0.03 | .04 |  |
| Asian American | $\pi_{004}$ | | -0.01 | | 0.06 | .00 | -0.01 | 0.06 | -.01 | -0.01 | 0.06 | -.01 |  |
| European American | $\pi_{005}$ | | -0.01 | | 0.05 | .00 | 0.00 | 0.05 | .00 | 0.01 | 0.05 | .00 |  |
| Different Ethnic Backgrounds | $\pi_{006}$ | | 0.15 | | 0.10 | .05 | 0.13 | 0.09 | .05 | 0.14 | 0.09 | .05 |  |
| Parental Education | $\pi_{007}$ | | 0.03* | | 0.01 | .08 | 0.03* | 0.01 | .08 | 0.03* | 0.01 | .08 |  |
| Age | $\gamma_{02k}$ | | -0.01 | | 0.01 | -.02 | 0.00 | 0.01 | .00 | -0.01 | 0.01 | -.02 |  |
| Previous Day’s Emotion | $\beta_{2jk}$ | | 0.10*** | | 0.02 | .08 | 0.10*** | 0.02 | .08 | 0.11*** | 0.01 | .08 |  |
| *Random Effects* | | | Variance | | *SE* |  | Variance | *SE* |  | Variance | *SE* |  |  |
| *Participant* | | |  | |  |  |  |  |  |  |  |  |  |
| Substance Use | | | 0.00 | | 0.00 |  | 0.00 | 0.00 |  | 0.01 | 0.01 |  |  |
| Age | | | 0.00 | | 0.00 |  | 0.00 | 0.00 |  | 0.00*** | 0.00 |  |  |
| Participant Intercept | | | 0.09*** | | 0.01 |  | 0.09*** | 0.01 |  | 0.08*** | 0.01 |  |  |
| *Wave* | | |  | |  |  |  |  |  |  |  |  |  |
| Arguments | | | 0.08*** | | 0.01 |  | 0.08*** | 0.01 |  | 0.08*** | 0.01 |  |  |
| Previous Day's Emotion | | | 0.04*** | | 0.01 |  | 0.04*** | 0.01 |  | 0.04*** | 0.01 |  |  |
| Wave Intercept | | | 0.08*** | | 0.01 |  | 0.08*** | 0.01 |  | 0.08*** | 0.01 |  |  |
| Arguments* Previous Day's Emotion | | | 0.00 | | 0.01 |  | 0.00 | 0.01 |  | 0.00 | 0.01 |  |  |
| Arguments*Wave Intercept | | | 0.02** | | 0.01 |  | 0.02** | 0.01 |  | 0.02*** | 0.01 |  |  |
| Previous Day's Emotion*Wave Intercept | | | 0.02*** | | 0.01 |  | 0.02*** | 0.01 |  | 0.02*** | 0.01 |  |  |
| Residual | | | 0.23*** | | 0.00 |  | 0.23*** | 0.00 |  | 0.23*** | 0.00 |  |  |

*Note*: Daily stressors were centered at the wave mean, substance use was centered at the grand mean, female was dummy-coded (male as reference group), ethnicity was dummy-coded (Latine as reference group), parents’ education was centered at the grand mean, age was centered at the grand mean, and previous day’s emotion was centered at the wave mean. **p*<.05, ***p*<.01, ****p*<.001.

*Table S7*. Summary of research questions and results.

| *Overarching Question* | *Operationalizing Emotion* | *Operationalizing Substance Use* | *Results* | *Arrow Representation of Results* |
| --- | --- | --- | --- | --- |
| How does substance use relate to emotional reactivity to stress? Do associations differ by sex? | How does substance use relate to **positive** emotional reactivity to stress? Do associations differ by sex? | Frequency of Alcohol Use | Main Effect: More frequent use is related to attenuated reactivity | _ꜜ_ male, _ꜜ_ female |
|  |  | Frequency of Cannabis Use | Main Effect: More frequent use is related to attenuated reactivity | _ꜜ_ male, _ꜜ_ female |
|  |  | Lifetime Substance Use | NS | — |
|  | How does substance use relate to **anxious** emotional reactivity to stress? Do associations differ by sex? | Frequency of Alcohol Use | In female adolescents, greater lifetime substance use is related to exaggerated reactivity | _ꜛ_ female |
|  |  | Frequency of Cannabis Use | NS | — |
|  |  | Lifetime Substance Use | In female adolescents, greater lifetime substance use is related to exaggerated reactivity | _ꜛ_ female |
|  | How does substance use relate to **depressive** emotional reactivity to stress? Do associations differ by sex? | Frequency of Alcohol Use | In male adolescents, more frequent alcohol use is related to attenuated reactivity | _ꜜ_ male |
|  |  | Frequency of Cannabis Use | In male adolescents, more frequent cannabis use is related to attenuated reactivity | _ꜜ_ male |
|  |  | Lifetime Substance Use | In male adolescents, greater lifetime substance use is related to attenuated reactivity | _ꜜ_ male |

_ꜛ_Indicates a significant negative association. _ꜜ_Indicates a significant negative association.

*n =* 4360 *M =* 14.2*, SD =* 2.4

*n =* 2979 *M =* 14.1*, SD =* 2.5

*n =* 2178 *M =* 14.1*, SD =* 2.7

Survey followed by 15 Daily Checklists (14 days plus practice day)

Survey followed by 15 Daily Checklists (14 days plus practice day)

Survey followed by 15 Daily Checklists (14 days plus practice day)

8

9

1

2

8

9

1

2

8

9

1

2


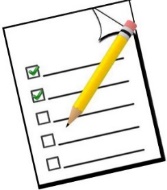


10

11

3

4

10

11

3

4

10

11

3

4


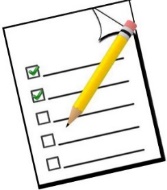

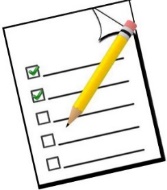


12

13

5

6

12

13

5

6

12

13

5

6

7

14

7

14

7

14

Wave 1

*n =* 307

Wave 2

*n =* 211

Wave 3

*n =* 154

2 Years

2 Years

2011-2012

10^th^ &11^th^ Grade

2013-2014
12^th^ Grade & 1Year
Post-High School

2015-2016
2 & 3 Years
Post-High School

*Figure S1*. Study schematic. *Note*: High levels of attrition were expected given that the study period includes the transition to college. Results of attrition analyses are provided in-text. The daily protocol period typically began one day after administration of the psychosocial survey. There were two weeks of checklists plus one practice day, which the majority of participants fully completed.

Poorer Emotion
(3 daily emotion outcomes)

3. Higher Depressive Emotion

Equation 2

Sex

2. Higher Anxious Emotion

1. Lower Positive Emotion

Degree of Daily Emotional Reactivity (Randomly Varying Daily Association)

Daily Arguments

Equation 1

Substance Use

(3 wave-level moderators)

1. Frequency of Alcohol Use

2. Frequency of Cannabis Use

3. Lifetime Substance Use

*Figure S2.* Statistical model of study research questions. *Note*: Positive emotion would be expected to decrease on days when adolescents experience more arguments (i.e., a negative coefficient), whereas anxious and depressive emotion would be expected to increase on days when adolescents experience more arguments (i.e., a positive coefficient). Therefore, a positive cross-level interaction would suggest that higher levels of substance use would be associated with lower positive emotional reactivity (i.e., a less negative daily association) and greater depressive and anxious emotional reactivity (i.e., a more positive daily association). In contrast, a negative cross-level interaction would suggest that higher levels of substance use would be associated with greater positive emotional reactivity (i.e., a more negative daily association) and lower depressive and anxious emotional reactivity (i.e., a less positive daily association).

*Simple Slopes*

$\beta_{1jk}$ = 0.19, *SE* = 0.04, *t*=4.38, 95% CI [0.10, 0.27], β=.12

$\beta_{1jk}$= 0.16, *SE* = 0.03, *t*=5.14, 95% CI [0.10, 0.22], β=.10

$\beta_{1jk}$= 0.13, *SE* = 0.04, *t*=3.33, 95% CI [0.05, 0.21], β=.09

Stressors

Stressors

Stressors

a)

b)

c)

*Simple Slopes*

$\beta_{1jk}$ = 0.17, *SE* = 0.05, *t*=3.82, 95% CI [0.08, 0.26], β=.11

$\beta_{1jk}$ = 0.16, *SE* = 0.03, *t*=5.11, 95% CI [0.10, 0.22], β=.10

$\beta_{1jk}$ = 0.14, *SE* = 0.06, *t*=2.57, 95% CI [0.04, 0.25], β=.09

*Simple Slopes*

$\beta_{1jk}$ = 0.18, *SE* = 0.04, 95% CI [0.09, 0.26], β=.11

$\beta_{1jk}$ = 0.16, *SE* = 0.03, 95% CI [0.10, 0.22], β=.10

$\beta_{1jk}$ = 0.15, *SE* = 0.04, 95% CI [0.08, 0.22], β=.09

Never Used Cannabis in the past year

Used Cannabis 3-11 days in the past year

Used Cannabis 2-3 days per month

Stressors

Stressors

Stressors

Stressors

Stressors

Stressors

*Figure S3.* Anxious Emotion as a Function of Daily Stressors and Frequency of Alcohol Use (a), Frequency of Cannabis Use (b), and Lifetime Substance Use (c) among male adolescents. *Note*: **p*<.05, ***p*<.01, ****p*<.001. SD = Standard Deviation. CI = Confidence Interval. Associations controlled for age, ethnicity, parents’ education, and previous day’s emotion. Frequency of alcohol use, frequency of cannabis use, and lifetime substance use are continuous variables, and associations were probed at approximately one standard deviation below the mean, the mean, and one standard deviation above the mean. All simple slopes are significant, *p*<.001, and do not significantly differ from one another.

Stressors

Stressors

Stressors

*Simple Slopes*

$\beta_{1jk}$ = 0.17, *SE* = 0.03, *t*=5.36, 95% CI [0.11, 0.24], β=.12

$\beta_{1jk}$ = 0.21, *SE* = 0.02, *t*=8.97, 95% CI [0.16, 0.26], β=.14

$\beta_{1jk}$ = 0.25, *SE* = 0.03, *t*=7.38, 95% CI [0.18, 0.32], β=.16

Stressors

Stressors

Stressors

*Simple Slopes*

$\beta_{1jk}$ = 0.20, *SE* = 0.03, *t*=7.67, 95% CI [0.15, 0.25], β=.13

$\beta_{1jk}$ = 0.22, *SE* = 0.03, *t*=7.88, 95% CI [0.17, 28], β=.15

$\beta_{1jk}$ = 0.25, *SE* = 0.05, *t*=5.06, 95% CI [0.15, 35], β=.17

Never Used Cannabis in the past year

Used Cannabis 3-11 days in the past year

Used Cannabis 2-3 days per month

Stressors

Stressors

Stressors

*Simple Slopes*

$\beta_{1jk}$ = 0.16, *SE* = 0.03, *t*=4.83, 95% CI [0.09, 0.22], β=.10

$\beta_{1jk}$ = 0.23, *SE* = 0.03, *t*=9.16, 95% CI [0.16, 0.25], β=.15

$\beta_{1jk}$ = 0.30, *SE* = 0.05, *t*=6.17, 95% CI [0.09, 0.22], β=.20

c)

b)

a)

c)

b)

*Figure S4.* Depressive Emotion as a Function of Daily Stressors and Frequency of Alcohol Use (a), Frequency of Cannabis Use (b), and Lifetime Substance Use (c) among female adolescents. *Note*: **p*<.05, ***p*<.01, ****p*<.001. SD = Standard Deviation. CI = Confidence Interval. Associations controlled for age, ethnicity, parents’ education, and previous day’s emotion. Frequency of alcohol use, frequency of cannabis use, and lifetime substance use are continuous variables. Associations were probed at approximately one standard deviation below the mean, the mean, and one standard deviation above the mean. All simple slopes are significant, *p*<.001, and do not significantly differ from one another.

*Study Materials*. Below are the measures for daily emotion, daily stressors, and substance use.

**The following is a list of feelings or experiences. How much did you experience them today?**

|  | *Not at all* | *A little* | *Moderately* | *Quite a bit* | *Extremely* |
| --- | --- | --- | --- | --- | --- |
| Attentive | 1 | 2 | 3 | 4 | 5 |
| Back, joint, or muscle pain | 1 | 2 | 3 | 4 | 5 |
| Calm | 1 | 2 | 3 | 4 | 5 |
| Cheerful | 1 | 2 | 3 | 4 | 5 |
| Discouraged | 1 | 2 | 3 | 4 | 5 |
| Enthusiastic | 1 | 2 | 3 | 4 | 5 |
| Excited | 1 | 2 | 3 | 4 | 5 |
| Exhausted | 1 | 2 | 3 | 4 | 5 |
| Fatigued | 1 | 2 | 3 | 4 | 5 |
| Frightened | 1 | 2 | 3 | 4 | 5 |
| Happy | 1 | 2 | 3 | 4 | 5 |
| Headache | 1 | 2 | 3 | 4 | 5 |
| Hopeless | 1 | 2 | 3 | 4 | 5 |
| Interested | 1 | 2 | 3 | 4 | 5 |
| Joyful | 1 | 2 | 3 | 4 | 5 |
| Nervous | 1 | 2 | 3 | 4 | 5 |
| On edge | 1 | 2 | 3 | 4 | 5 |
| Sad | 1 | 2 | 3 | 4 | 5 |
| Scared | 1 | 2 | 3 | 4 | 5 |
| Sleepy | 1 | 2 | 3 | 4 | 5 |
| Threatened | 1 | 2 | 3 | 4 | 5 |
| Tired | 1 | 2 | 3 | 4 | 5 |
| Trouble sleeping | 1 | 2 | 3 | 4 | 5 |
| Unable to concentrate | 1 | 2 | 3 | 4 | 5 |
| Uneasy | 1 | 2 | 3 | 4 | 5 |
| Unsafe | 1 | 2 | 3 | 4 | 5 |
| Worn-out | 1 | 2 | 3 | 4 | 5 |
| Worried | 1 | 2 | 3 | 4 | 5 |

**Stressors**

**Did any of the following activities or events occur to you today? (check if YES)**

Oargued with your mother or father about something

Oargued with another family member about something

Ohad an argument or were punished by an adult at school

Ohad an argument with a close friend or partner

**Substance Use**

1. If you have ever smoked more than one or two puffs of a cigarette, how old were you when you smoked that much for the first time?

I have never smoked more than one or two puffs of a cigarette

Less than 9 years old

9 or 10 years old

11 or 12 years old

13 or 14 years old

15 or 16 years old

17 years old or older

2. If you have ever had more than a few sips of alcohol, how old were you when you first drank that much alcohol?

I have never had a drink of alcohol other than a few sips

Less than 9 years old

9 or 10 years old

11 or 12 years old

13 or 14 years old

15 or 16 years old

17 years old or older

3. If you have ever tried marijuana (pot, weed, grass, hash, etc.), how old were you when you tried it for the first time?

I have never tried marijuana

Less than 9 years old

9 or 10 years old

11 or 12 years old

13 or 14 years old

15 or 16 years old

17 years old or older

4. If you have ever tried any form of cocaine, including powder, crack, or freebase, how old were you when you tried it for the first time?

I have never tried cocaine

Less than 9 years old

9 or 10 years old

11 or 12 years old

13 or 14 years old

15 or 16 years old

17 years old or older

5. If you have ever used crystal meth (also called "ice" or “glass”), how old were you when you used crystal meth for the first time?

I have never tried crystal meth

Less than 9 years old

9 or 10 years old

11 or 12 years old

13 or 14 years old

15 or 16 years old

17 years old or older

6. If you have ever used any other type of illegal drug, such as LSD, PCP, ecstasy, mushrooms, speed, or heroin, how old were you when you used them for the first time?

I have never tried any other illegal drugs

Less than 9 years old

9 or 10 years old

11 or 12 years old

13 or 14 years old

15 or 16 years old

17 years old or older

7. If you have ever used any prescription drug such Ritalin, oxycotin, adderall, a valium, any narcotic, or any tranquilizer without a prescription, how old were you when you used a prescription drug without a prescription for the first time?

I have never used prescription drugs

Less than 9 years old

9 or 10 years old

11 or 12 years old

13 or 14 years old

15 or 16 years old

17 years old or older

Syntax for Analyses

*All analyses were tested in Stata 16.1

*Descriptives

sum Mean_PositiveMean Mean_DepressiveMean Mean_AnxiousMean Mean_ArgueMean Mean_SubCount Mean_AlcYear Mean_PotYear gender aEthnicity_W123 ParentEdu Age gender if Wave==1 & Day==2 & Participation_Subject==1

sum Mean_PositiveMean Mean_DepressiveMean Mean_AnxiousMean Mean_ArgueMean Mean_SubCount Mean_AlcYear Mean_PotYear gender aEthnicity_W123 ParentEdu aAge_W gender if Wave==1 & Day==2 & Participation_Subject==1

sum TotalDays if Participation_Subject==1 & Wave==1 & Day==1

tab TotalWaves if Day==1 & Wave==1

gen PercentagePossibleWaves=WaveTotal/3 if SubjectID<6000

replace PercentagePossibleWaves=WaveTotal/2 if SubjectID>5999

*Participation Analyses

ttest PercentagePossibleWaves if Wave==1 & Day==1 & Participation_Subject==1, by(gender)

anova PercentagePossibleWaves aEthnicity_W123 if Wave==1 & Day==1 & Participation_Subject==1

tabstat PercentagePossibleWaves if Wave==1 & Day==1 & Participation_Subject==1, by(aEthnicity_W123)

pwmean PercentagePossibleWaves if Wave==1 & Day==1 & Participation_Subject==1, over(aEthnicity_W123)

**Differences at study entry

gen Age_forentry=aAge_W if Participation_Wave==1

by SubjectID: egen age_at_entry=min(Age_forentry) if Participation_Wave==1

gen SubCount_forentry=SubCount if Participation_Wave==1

by SubjectID: egen SubCount_at_entry=min(SubCount_forentry) if Participation_Wave==1

gen AlcYear_forentry=AlcYear if Participation_Wave==1

by SubjectID: egen AlcYear_at_entry=min(AlcYear_forentry) if Participation_Wave==1

gen PotYear_forentry=PotYear if Participation_Wave==1

by SubjectID: egen PotYear_at_entry=min(PotYear_forentry) if Participation_Wave==1

pwcorr PercentagePossibleWaves Mean_PositiveMean Mean_DepressiveMean Mean_AnxiousMean Mean_ArgueMean SubCount_at_entry PotYear_forentry AlcYear_forentry age_at_entry ParentEdu if Wave==1 & Day==1 & Participation_Subject==1, o sig

*Multilevel Participation Models

mixed SubCount PercentagePossibleWaves Age_c || SubjectID: if Participation_Subject==1 & day==1, var cov()

mixed PotYear PercentagePossibleWaves Age_c || SubjectID: if Participation_Subject==1 & day==1, var cov()

mixed AlcYear PercentagePossibleWaves Age_c || SubjectID: if Participation_Subject==1 & day==1, var cov()

mixed positivemean PercentagePossibleWaves Age_c || SubjectID: || Wave: if Participation_Subject==1, var cov()

mixed depressivemean PercentagePossibleWaves Age_c || SubjectID: ||Wave: if Participation_Subject==1, var cov()

mixed anxiousmean PercentagePossibleWaves Age_c || SubjectID: ||Wave: if Participation_Subject==1, var cov()

mixed arguesum PercentagePossibleWaves Age_c || SubjectID: ||Wave: if Participation_Subject==1 , var cov()

*Compare Participants in Analytic vs Full Sample

gen Participation_Subject_di= Participation_Subject

replace Participation_Subject_di=0 if Participation_Subject==.

tab gender Participation_Subject_di if Wave==1 & Day==1, ch

tab aEthnicity_W123 Participation_Subject_di if Wave==1 & Day==1, ch

ttest ParentEdu if Wave==1 & Day==1, by(Participation_Subject_di)

mixed SubCount Participation_Subject_di Age_c || SubjectID: if day==1, var cov()

mixed PotYear Participation_Subject_di Age_c || SubjectID: if day==1, var cov()

mixed AlcYear Participation_Subject_di Age_c || SubjectID: if day==1, var cov()

mixed positivemean Participation_Subject_di Age_c || SubjectID: || Wave: , var cov()

mixed depressivemean Participation_Subject_di Age_c || SubjectID: ||Wave: , var cov()

mixed anxiousmean Participation_Subject_di Age_c || SubjectID: ||Wave: , var cov()

mixed arguesum Participation_Subject_di Age_c || SubjectID: ||Wave: , var cov()

*Descriptive Emotion Reactivity Models

mixed anxiousmean c.Argue_mc i.gender ib3.aEthnicity_W123 ParentEdu_c Age_c anxious_lag_c || SubjectID: Age_c || Wave: Argue_mc anxious_lag_c, var cov(unstr)

estimates store DescriptiveAnx

mixed depressivemean c.Argue_mc i.gender ib3.aEthnicity_W123 ParentEdu_c Age_c depressive_lag_c || SubjectID: Age_c || Wave: Argue_mc depressive_lag_c, var cov(unstr)

estimates store DescriptiveDep

mixed positivemean c.Argue_mc i.gender ib3.aEthnicity_W123 ParentEdu_c Age_c positive_lag_c || SubjectID: Age_c || Wave: Argue_mc positive_lag_c, var cov(unstr)

estimates store DescriptivePos

*Primary models: Associations between Substance Use and Emotional Reactivity (Tables 1-3)

mixed positivemean c.Argue_mc##c.SubCount_mc gender ib3.aEthnicity_W123 ParentEdu_c Age_c positive_lag_c || SubjectID: Age_c SubCount_mc || Wave:Argue_mc positive_lag_c, var cov(unstr)

estimates store pos1

mixed positivemean c.Argue_mc##c.AlcYear_mc gender ib3.aEthnicity_W123 ParentEdu_c Age_c positive_lag_c || SubjectID: Age_c AlcYear_mc || Wave: Argue_mc positive_lag_c, var cov(unstr)

estimates store pos2

mixed positivemean c.Argue_mc##c.PotYear_mc gender ib3.aEthnicity_W123 ParentEdu_c Age_c positive_lag_c || SubjectID: Age_c PotYear_mc || Wave: Argue_mc positive_lag_c, var cov(unstr)

estimates store pos3

mixed anxiousmean c.Argue_mc##c.SubCount_mc##gender ib3.aEthnicity_W123 ParentEdu_c Age_c anxious_lag_c || SubjectID: Age_c SubCount_mc || Wave: Argue_mc anxious_lag_c, var cov(unstr)

estimates store anx1

mixed anxiousmean c.Argue_mc##c.AlcYear_mc##gender ib3.aEthnicity_W123 ParentEdu_c Age_c anxious_lag_c || SubjectID: Age_c AlcYear_mc || Wave: Argue_mc anxious_lag_c, var cov(unstr)

estimates store anx2

mixed anxiousmean c.Argue_mc##c.PotYear_mc##gender ib3.aEthnicity_W123 ParentEdu_c Age_c anxious_lag_c || SubjectID: Age_c PotYear_mc || Wave: Argue_mc anxious_lag_c, var cov(unstr)

estimates store anx3

mixed depressivemean c.Argue_mc##c.SubCount_mc##gender ib3.aEthnicity_W123 ParentEdu_c Age_c depressive_lag_c || SubjectID: Age_c SubCount_mc || Wave: Argue_mc depressive_lag_c, var cov(unstr)

estimates store dep1

mixed depressivemean c.Argue_mc##c.AlcYear_mc##gender ib3.aEthnicity_W123 ParentEdu_c Age_c depressive_lag_c || SubjectID: Age_c AlcYear_mc || Wave: Argue_mc depressive_lag_c, var cov(unstr)

estimates store dep2

mixed depressivemean c.Argue_mc##c.PotYear_mc##gender ib3.aEthnicity_W123 ParentEdu_c Age_c depressive_lag_c || SubjectID: Age_c PotYear_mc || Wave: Argue_mc depressive_lag_c, var cov(unstr)

estimates store dep3

estout DescriptivePos DescriptiveAnx DescriptiveDep , cells((b(star fmt(2)) se(par fmt(2))))

estout pos1 pos2 pos3 , cells((b(star fmt(2)) se(par fmt(2))))

estout anx1 anx2 anx3 , cells((b(star fmt(2)) se(par fmt(2))))

estout dep1 dep2 dep3 , cells((b(star fmt(2)) se(par fmt(2))))

*Follow-up analyses covarying emotion variability and mean number of arguments at that wave

**Calculating emotion variability

sort IDWave Day

by IDWave: egen PositiveSD=sd(positivemean)

by IDWave: egen AnxiousSD=sd(anxiousmean)

by IDWave: egen DepressiveSD=sd(depressivemean)

*Analyses (primary models with additional covariates)

mixed positivemean c.Argue_mc##c.SubCount_mc gender ib3.aEthnicity_W123 ParentEdu_c Age_c positive_lag_c PositiveSD Mean_ArgueMean || SubjectID: Age_c SubCount_mc || Wave:Argue_mc positive_lag_c, var cov(unstr)

mixed positivemean c.Argue_mc##c.AlcYear_mc gender ib3.aEthnicity_W123 ParentEdu_c Age_c positive_lag_c PositiveSD Mean_ArgueMean || SubjectID: Age_c AlcYear_mc || Wave: Argue_mc positive_lag_c, var cov(unstr)

mixed positivemean c.Argue_mc##c.PotYear_mc gender ib3.aEthnicity_W123 ParentEdu_c Age_c positive_lag_c PositiveSD Mean_ArgueMean || SubjectID: Age_c PotYear_mc || Wave: Argue_mc positive_lag_c, var cov(unstr)

mixed anxiousmean c.Argue_mc##c.SubCount_mc##gender ib3.aEthnicity_W123 ParentEdu_c Age_c anxious_lag_c AnxiousSD Mean_ArgueMean || SubjectID: Age_c SubCount_mc || Wave: Argue_mc anxious_lag_c, var cov(unstr)

mixed anxiousmean c.Argue_mc##c.AlcYear_mc##gender ib3.aEthnicity_W123 ParentEdu_c Age_c anxious_lag_c AnxiousSD Mean_ArgueMean || SubjectID: Age_c AlcYear_mc || Wave: Argue_mc anxious_lag_c, var cov(unstr)

mixed anxiousmean c.Argue_mc##c.PotYear_mc##gender ib3.aEthnicity_W123 ParentEdu_c Age_c anxious_lag_c AnxiousSD Mean_ArgueMean || SubjectID: Age_c PotYear_mc || Wave: Argue_mc anxious_lag_c, var cov(unstr)

mixed depressivemean c.Argue_mc##c.SubCount_mc##gender ib3.aEthnicity_W123 ParentEdu_c Age_c depressive_lag_c DepressiveSD Mean_ArgueMean || SubjectID: Age_c SubCount_mc PositiveSD Mean_ArgueMean || Wave: Argue_mc depressive_lag_c, var cov(unstr)

mixed depressivemean c.Argue_mc##c.AlcYear_mc##gender ib3.aEthnicity_W123 ParentEdu_c Age_c depressive_lag_c DepressiveSD Mean_ArgueMean || SubjectID: Age_c AlcYear_mc || Wave: Argue_mc depressive_lag_c, var cov(unstr)

mixed depressivemean c.Argue_mc##c.PotYear_mc##gender ib3.aEthnicity_W123 ParentEdu_c Age_c depressive_lag_c DepressiveSD Mean_ArgueMean || SubjectID: Age_c PotYear_mc || Wave: Argue_mc depressive_lag_c, var cov(unstr)

*Likelihood ratio tests to determine whether age and substance use should be treated as random effects in primary models

mixed anxiousmean c.Argue_mc##c.SubCount_mc##gender i.aEthnicity_W123 ParentEdu_c Age_c anxious_lag_c || SubjectID: || Wave: Argue_mc anxious_lag_c, var cov(unstr)

estimates store anx1

mixed anxiousmean c.Argue_mc##c.SubCount_mc##gender i.aEthnicity_W123 ParentEdu_c Age_c anxious_lag_c || SubjectID: SubCount_mc || Wave: Argue_mc anxious_lag_c, var cov(unstr)

estimates store anx1sub

mixed anxiousmean c.Argue_mc##c.SubCount_mc##gender i.aEthnicity_W123 ParentEdu_c Age_c anxious_lag_c || SubjectID: Age_c || Wave: Argue_mc anxious_lag_c, var cov(unstr)

estimates store anx1age

lrtest anx1 anx1sub, stats

lrtest anx1 anx1age, stats

mixed anxiousmean c.Argue_mc##c.AlcYear_mc##gender i.aEthnicity_W123 ParentEdu_c Age_c anxious_lag_c || SubjectID: || Wave: Argue_mc anxious_lag_c, var cov(unstr)

estimates store anx2

mixed anxiousmean c.Argue_mc##c.AlcYear_mc##gender i.aEthnicity_W123 ParentEdu_c Age_c anxious_lag_c || SubjectID: AlcYear_mc || Wave: Argue_mc anxious_lag_c, var cov(unstr)

estimates store anx2sub

mixed anxiousmean c.Argue_mc##c.AlcYear_mc##gender i.aEthnicity_W123 ParentEdu_c Age_c anxious_lag_c || SubjectID: Age_c || Wave: Argue_mc anxious_lag_c, var cov(unstr)

estimates store anx2age

lrtest anx2 anx2sub, stats

lrtest anx2 anx2age, stats

mixed anxiousmean c.Argue_mc##c.PotYear_mc##gender i.aEthnicity_W123 ParentEdu_c Age_c anxious_lag_c || SubjectID: || Wave: Argue_mc anxious_lag_c, var cov(unstr)

estimates store anx3

mixed anxiousmean c.Argue_mc##c.PotYear_mc##gender i.aEthnicity_W123 ParentEdu_c Age_c anxious_lag_c || SubjectID: PotYear_mc || Wave: Argue_mc anxious_lag_c, var cov(unstr)

estimates store anx3sub

mixed anxiousmean c.Argue_mc##c.PotYear_mc##gender i.aEthnicity_W123 ParentEdu_c Age_c anxious_lag_c || SubjectID: Age_c || Wave: Argue_mc anxious_lag_c, var cov(unstr)

estimates store anx3age

lrtest anx3 anx3sub, stats

lrtest anx3 anx3age, stats

mixed depressivemean c.Argue_mc##c.SubCount_mc##gender i.aEthnicity_W123 ParentEdu_c Age_c depressive_lag_c || SubjectID: || Wave: Argue_mc depressive_lag_c, var cov(unstr)

estimates store dep1

mixed depressivemean c.Argue_mc##c.SubCount_mc##gender i.aEthnicity_W123 ParentEdu_c Age_c depressive_lag_c || SubjectID: SubCount_mc || Wave: Argue_mc depressive_lag_c, var cov(unstr)

estimates store dep1sub

mixed depressivemean c.Argue_mc##c.SubCount_mc##gender i.aEthnicity_W123 ParentEdu_c Age_c depressive_lag_c || SubjectID: Age_c || Wave: Argue_mc depressive_lag_c, var cov(unstr)

estimates store dep1age

lrtest dep1 dep1sub, stats

lrtest dep1 dep1age, stats

mixed depressivemean c.Argue_mc##c.AlcYear_mc##gender i.aEthnicity_W123 ParentEdu_c Age_c depressive_lag_c || SubjectID: || Wave: Argue_mc depressive_lag_c, var cov(unstr)

estimates store dep2

mixed depressivemean c.Argue_mc##c.AlcYear_mc##gender i.aEthnicity_W123 ParentEdu_c Age_c depressive_lag_c || SubjectID: AlcYear_mc || Wave: Argue_mc depressive_lag_c, var cov(unstr)

estimates store dep2sub

mixed depressivemean c.Argue_mc##c.AlcYear_mc##gender i.aEthnicity_W123 ParentEdu_c Age_c depressive_lag_c || SubjectID: Age_c || Wave: Argue_mc depressive_lag_c, var cov(unstr)

estimates store dep2age

lrtest dep2 dep2sub, stats

lrtest dep2 dep2age, stats

mixed depressivemean c.Argue_mc##c.PotYear_mc##gender i.aEthnicity_W123 ParentEdu_c Age_c depressive_lag_c || SubjectID: || Wave: Argue_mc depressive_lag_c, var cov(unstr)

estimates store dep3

mixed depressivemean c.Argue_mc##c.PotYear_mc##gender i.aEthnicity_W123 ParentEdu_c Age_c depressive_lag_c || SubjectID: PotYear_mc || Wave: Argue_mc depressive_lag_c, var cov(unstr)

estimates store dep3sub

mixed depressivemean c.Argue_mc##c.PotYear_mc##gender i.aEthnicity_W123 ParentEdu_c Age_c depressive_lag_c || SubjectID: Age_c || Wave: Argue_mc depressive_lag_c, var cov(unstr)

estimates store dep3age

lrtest dep3 dep3sub, stats

lrtest dep3 dep3age, stats

mixed positivemean c.Argue_mc##c.SubCount_mc gender i.aEthnicity_W123 ParentEdu_c Age_c positive_lag_c || SubjectID: || Wave:Argue_mc positive_lag_c, var cov(unstr)

estimates store pos1

mixed positivemean c.Argue_mc##c.SubCount_mc gender i.aEthnicity_W123 ParentEdu_c Age_c positive_lag_c || SubjectID: SubCount_mc || Wave:Argue_mc positive_lag_c, var cov(unstr)

estimates store pos1sub

mixed positivemean c.Argue_mc##c.SubCount_mc gender i.aEthnicity_W123 ParentEdu_c Age_c positive_lag_c || SubjectID: Age_c || Wave:Argue_mc positive_lag_c, var cov(unstr)

estimates store pos1age

lrtest pos1 pos1sub, stats

lrtest pos1 pos1age, stats

mixed positivemean c.Argue_mc##c.AlcYear_mc gender i.aEthnicity_W123 ParentEdu_c Age_c positive_lag_c || SubjectID: || Wave:Argue_mc positive_lag_c, var cov(unstr)

estimates store pos2

mixed positivemean c.Argue_mc##c.AlcYear_mc gender i.aEthnicity_W123 ParentEdu_c Age_c positive_lag_c || SubjectID: AlcYear_mc || Wave:Argue_mc positive_lag_c, var cov(unstr)

estimates store pos2sub

mixed positivemean c.Argue_mc##c.AlcYear_mc gender i.aEthnicity_W123 ParentEdu_c Age_c positive_lag_c || SubjectID: Age_c || Wave:Argue_mc positive_lag_c, var cov(unstr)

estimates store pos2age

lrtest pos2 pos2sub, stats

lrtest pos2 pos2age, stats

mixed positivemean c.Argue_mc##c.PotYear_mc gender i.aEthnicity_W123 ParentEdu_c Age_c positive_lag_c || SubjectID: || Wave:Argue_mc positive_lag_c, var cov(unstr)

estimates store pos3

mixed positivemean c.Argue_mc##c.PotYear_mc gender i.aEthnicity_W123 ParentEdu_c Age_c positive_lag_c || SubjectID: PotYear_mc || Wave:Argue_mc positive_lag_c, var cov(unstr)

estimates store pos3sub

mixed positivemean c.Argue_mc##c.PotYear_mc gender i.aEthnicity_W123 ParentEdu_c Age_c positive_lag_c || SubjectID: Age_c || Wave:Argue_mc positive_lag_c, var cov(unstr)

estimates store pos3age

lrtest pos3 pos3sub, stats

lrtest pos3 pos3age, stats
